# Gold-Nanoparticle Hybrid Nanostructures for Multimodal Cancer Therapy

**DOI:** 10.3390/nano12203706

**Published:** 2022-10-21

**Authors:** Amaal Abdulraqeb Ali, Waad H. Abuwatfa, Mohammad H. Al-Sayah, Ghaleb A. Husseini

**Affiliations:** 1 Biomedical Engineering Graduate Program, College of Engineering, American University of Sharjah, Sharjah P.O. Box 26666, United Arab Emirates; 2 Department of Chemical Engineering, College of Engineering, American University of Sharjah, Sharjah P.O. Box 26666, United Arab Emirates; 3 Materials Science and Engineering Program, College of Arts and Sciences, American University of Sharjah, Sharjah P.O. Box 26666, United Arab Emirates; 4 Department of Biology, Chemistry and Environmental Sciences, American University of Sharjah, Sharjah P.O. Box 26666, United Arab Emirates

**Keywords:** gold-nanoparticle hybrid nanostructures, multimodal therapy, photothermal therapy, triggered drug delivery

## Abstract

With the urgent need for bio-nanomaterials to improve the currently available cancer treatments, gold nanoparticle (GNP) hybrid nanostructures are rapidly rising as promising multimodal candidates for cancer therapy. Gold nanoparticles (GNPs) have been hybridized with several nanocarriers, including liposomes and polymers, to achieve chemotherapy, photothermal therapy, radiotherapy, and imaging using a single composite. The GNP nanohybrids used for targeted chemotherapy can be designed to respond to external stimuli such as heat or internal stimuli such as intratumoral pH. Despite their promise for multimodal cancer therapy, there are currently no reviews summarizing the current status of GNP nanohybrid use for cancer theragnostics. Therefore, this review fulfills this gap in the literature by providing a critical analysis of the data available on the use of GNP nanohybrids for cancer treatment with a specific focus on synergistic approaches (i.e., triggered drug release, photothermal therapy, and radiotherapy). It also highlights some of the challenges that hinder the clinical translation of GNP hybrid nanostructures from bench to bedside. Future studies that could expedite the clinical progress of GNPs, as well as the future possibility of improving GNP nanohybrids for cancer theragnostics, are also summarized.

## 1. Introduction

A wide range of bio-nanomaterials is becoming a subject of interest for biomedical purposes. Of those nanomaterials, FDA-approved gold nanoparticles have been well-studied for their promising role in improving drug delivery and imaging [1,2,3]. Gold nanoparticles (GNPs), which are composed of gold atom aggregates of sizes ranging from 1 to 100 nm [4], have been extensively studied and utilized for biomedical applications, including the diagnosis and/or treatment of cancer [5,6], among others [7,8]. This is mainly due to their unique localized surface plasmon resonance (LSPR) and photothermal conversion ability, as reviewed by Vines et al. [9] and Sztandera et al. [10]. LSPR results when nanoparticles are irradiated with light of a particular wavelength, causing the surface electrons in the metal conduction band to oscillate coherently, resulting in the separation of their surface charge (dipole oscillation) [9,11]. Although all noble metal nanoparticles exhibit LSPR, GNPs are classified as the most stable, rendering them advantageous over other LSPR-characterized nanoparticles [12].

Stemming from their LSPR property, GNPs possess the ability to convert light (i.e., near-infrared (NIR) light) to heat in a process known as photothermal conversion. Photothermal conversion makes GNPs suitable candidates for the thermal ablation of cancer cells in a noninvasive treatment strategy known as photothermal therapy (PTT) [9,13]. Eradicating tumor cells via heat is especially advantageous in cancer therapy due to cells’ higher sensitivity to heat compared with normal ones [14]. Furthermore, heat generation was reported to intensify chemotherapeutic cytotoxic effects by increasing the blood vessel permeability, thereby allowing more drugs to reach and accumulate at the tumor site. Heat can also trigger the release of encapsulated drugs from heat-sensitive carriers, thereby achieving more tumor-specific drug release and avoiding drug-associated, off-target, unwanted side effects [15,16,17,18]. Although other nanomaterials, such as magnetic nanoparticles, can induce hyperthermia, GNP-associated photothermal conversion provides practical advantages over other nanomaterials. For instance, magnetic nanoparticles require the application of an alternating magnetic field to the whole body to trigger heat generation. In contrast, GNP photothermal conversion involves the application of a near-infrared (NIR) laser specifically to the site of interest rather than to the whole body [9]. Furthermore, GNPs were found to be relatively safer than other metal nanoparticles [19], with a safety profile that depends on several factors, including size, shape [9], and concentration [20]. 

Moreover, GNPs’ various possible sizes, shapes, and surface functionalizations provide a level of control over the nanoparticles and allow further tailoring of their properties for specific applications to be conducted [21]. For instance, Yang et al. [22] reported that gold nanostars were found to possess higher photothermal conversion abilities than spherical or rod-shaped GNPs. In contrast, spherical GNPs showed higher uptake by cells compared to gold nanorods. Chan et al. reported that the size of spherical GNPs also influenced their uptake levels, with the highest degree of uptake being achieved for a size of 50 nm [23]. Furthermore, GNPs also serve as efficient radiosensitizing agents due to their high atomic number and ability to absorb X-rays, which makes them good candidates for tumor radiosensitization [24]. Their strong X-ray absorption abilities make them suitable as computed tomography (CT) contrast agents [25]. In fact, GNPs were reported to improve radiotherapy [24,26,27] and CT imaging [25,28,29]. Hence, GNPs can provide a multimodal therapeutic platform capable of chemotherapy delivery, PTT, radiotherapy, and imaging.

**Figure 1:**
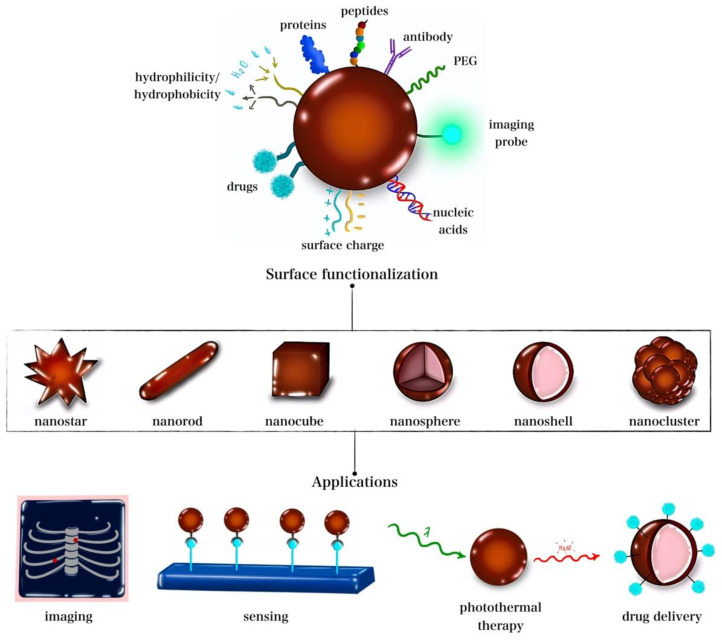
GNP hybrid nanostructures shapes, functionalization, and common applications.

Multimodal therapeutic platforms have been explored to overcome tumor resistance to chemo-radiotherapy. Tumors are known to develop resistance to both chemotherapy and radiotherapy, rendering them eventually ineffective. Therefore, combining chemotherapy/radiotherapy with the hyperthermic annihilation of cancer cells could combat chemotherapy-/radiotherapy-resistant tumors. However, the combination of chemotherapy, radiotherapy, and PTT poses another clinical challenge, as it exposes the patient to a higher level of toxicity [24]. Such a challenge could be overcome with nanoparticles to achieve chemo-radiotherapy and PTT. This is due to the nanoparticle ability to preferentially accumulate at the tumor site due to the enhanced permeability and retention (EPR) effect, thereby leaving normal tissues with tightly junctioned blood vessels more or less void of nanoparticles [30]. Figure 1 summarizes the different shapes, surface engineering, functionalization moieties, and some common theragnostic applications of GNPs.

Despite the extensive advances in utilizing nanomaterials, including GNPs, for biomedical applications, individual nanomaterials still suffer from limitations of their own. For instance, systematically administered PTT materials such as inorganic nanoparticles tend to accumulate mostly in the liver and spleen rather than at the tumor site, thereby limiting their therapeutic effectivity. When administered directly to the tumor site to avoid liver and spleen accumulation, nanomaterials are prone to be rapidly cleared up due to their small size. Additionally, cancer treatment usually requires multiple, repeated treatments, which could be difficult with such rapidly cleared, unretained nanoparticles. Furthermore, those inorganic PTT nanomaterials are usually nondegradable [31]. To overcome such limitations, one well-developed strategy is the hybridization of nanomaterials to develop nanostructures with combined advantages and/or compensated weaknesses. Such hybridized nanocomposites are designed to have a performance surpassing that of their individual components [32]. Among these are GNP nanohybrids, which are rapidly emerging as promising candidates for cancer therapy via dual PTT and the triggered delivery of chemotherapeutics. Some GNP hybrid nanostructures were reported to prolong circulation time and increase their cellular internalization rate compared with conventional GNPs, thus achieving more effective and specific delivery of the carried drugs [33]. Furthermore, GNP nanohybrids can also achieve thermoresponsive drug release when combined with a heat-sensitive nanocarrier [34,35,36]. 

GNPs hybridized with stimuli-sensitive nanocarriers for triggered drug release, individually or combined with other approaches for synergistic (e.g., combined chemotherapy and hyperthermia), multimodal tumor cell ablation, are becoming an increasingly explored topic. For example, GNP photothermal conversion abilities were combined with nanocarriers that responded to heat and other conditions of the tumor microenvironment (TME) [37]. Such unique conditions (e.g., low pH) in a hybrid nanostructure allow a higher degree of tumor-specificity and improved cancer treatment to be obtained [38,39]. 

Therefore, hybridizing GNPs with other nanocarriers can overcome the limitations associated with conventional GNPs, such as avoiding liver and spleen accumulation, rapid clearance, and higher tumor specificity [31], thereby making them viable candidates for cancer therapy. While the use of GNPs for multimodal cancer therapy has been well investigated, with several reviews summarizing their potential and progress in the field [9,40,41], GNP nanohybrids remain relatively newly studied nanocomposites, with no current reviews summarizing the status of their use as cancer multimodal therapeutic platforms. This review fulfills this gap in the literature by discussing and critically analyzing recent research on the use of GNP hybrid nanostructures for multimodal cancer therapy while focusing on the synergistic approaches involving GNP-related features (e.g., heat-triggered drug release and PTT). This article also discusses the challenges hindering the further progress of GNP nanohybrids from the lab bench to the patient bedside, and future directions to facilitate their progress.

## 2. Smart Drug Delivery Nanocarriers

Several treatment strategies have been developed to combat the disease, including the most commonly utilized approach, chemotherapy. However, despite the advances achieved, cancer therapeutics still possess major limitations that restrict their use. Therefore, interest has shifted towards exploiting nano-based approaches, which hold the potential to overcome those limitations [42]. Chemotherapy is considered one of the most effective cancer treatments available, whether as a single treatment modality or combined with other approaches. However, chemotherapy is limited by its inability to discriminate between cancerous and normal cells, resulting in off-target toxicities [42]. In addition to systematic toxicity, some approved cancer therapeutics also suffer from poor water solubility and a short circulation half-life [43]. Such side effects and limitations can be overcome by trapping the drug within a nanosized carrier capable of carrying the drug through biological barriers to the tumor site and releasing the drug when triggered [43,44,45,46].

Drug nanocarriers have been developed and studied extensively for cancer therapy using a variety of carriers and drugs. Nanocarriers could be used to allow the delivery of a drug across some of the highly selective biological barriers, such as the blood–brain barrier (BBB) [47]. In addition, several nanocarriers possess stimulus responsiveness due to the structural changes they undergo in response to particular stimuli, such as pH, temperature [48,49], or redox [48], which can be utilized to achieve tailored drug release. Due to their specificity in release, such “smart” nanovehicles for drug delivery purposes have become a widely investigated and reported strategy in the literature [46,47,48,49,50,51]. 

**Figure 2:**
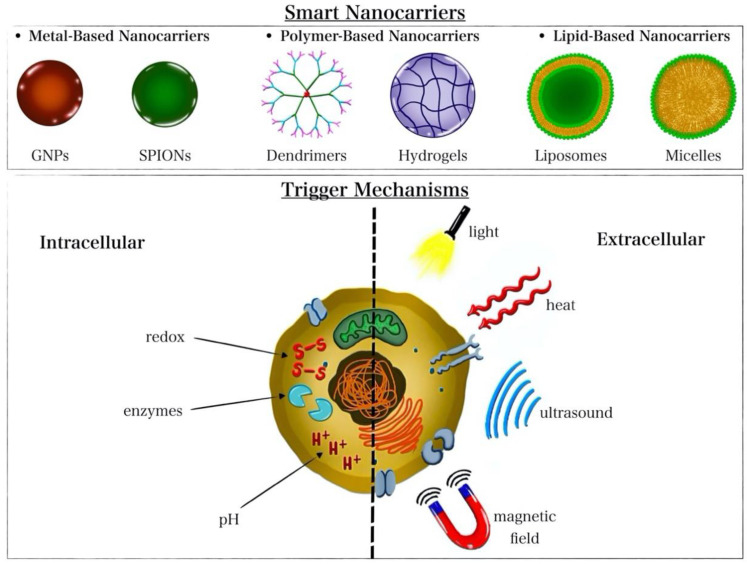
Illustration of the different nanocarrier types and release-triggering mechanisms.

Some of the most explored nanocarriers for drug delivery purposes are liposomes, micelles, hydrogels, GNPs, iron oxide nanoparticles, carbon-based nanomaterials (e.g., carbon nanotubes), mesoporous nanoparticles, and dendrimers. Different nanocarriers utilize different structures, drug encapsulation mechanisms, and release-triggering stimuli [52,53]. Generally, nanoparticle-mediated delivery enhances drug solubility, bioavailability, stability, and circulation time while reducing its side effects. Broadly, nanocarriers can be divided into metal-based, polymeric, and lipid-based nanocarriers:

(1) Metal-based nanocarriers are among the emerging materials for biomedicine and drug delivery applications [54]. GNPs and iron oxide nanoparticles (IONPs) have been increasingly studied for drug delivery purposes, as reviewed by Hossen et al. [53]. GNPs and IONPs share the common attractive feature of heat generation that can trigger drug release and/or kill cells via thermal ablation. Both nanoparticles have the benefits of easy synthesis and surface functionalization, [53] and serve as contrast agents to enhance imaging and achieve image-guided therapy [55,56,57]. Additionally, SPIONs exhibit the advantageous property of magnetic targeting via an external magnetic field for spatial targeting [58]. Venditti et al. reported that GNPs are used to improve the bioavailability of drugs [59]. Yet, the practical application of such metal-based nanocarriers can be limited by their potential toxicity [60];
(2) Polymer-based smart nanocarriers include hydrogels and dendrimers. Dendrimers are large, highly branched polymers capable of loading drugs via entrapment in spaces within the network or by attaching to branching points (via hydrogen bonding or to surface groups via electrostatic interactions) [61,62]. Hydrogels, on the other hand, are composed of hydrophilic crosslinked polymer chains capable of cargo entrapment and delivery [63,64,65]. Dendrimers and hydrogels have been reported for the efficient delivery of genes, drugs, and proteins [66,67,68,69,70,71] and for stimulus-responsive release under various triggers, including temperature, pH, and redox conditions [72,73]. However, dendrimers suffer from their complicated and costly synthesis procedures, and both dendrimers and hydrogels are restricted by their ability to host solely hydrophilic drugs [60];
(3) Lipid-based nanocarriers include liposomes and micelles. Liposomes, membrane-like self-assembled lipid bilayers, are utilized for the delivery of hydrophobic/hydrophilic drugs, genes, and proteins while possessing high biocompatibility and stimulus responsiveness (e.g., ultrasound and temperature responsiveness) [74]. Micelles are organic nanocarriers similar in structure to liposomes but made up of a single layer. Unlike liposomes, micelles can also be composed of amphiphilic polymers [75,76]. Micelles are used to transport hydrophobic drugs, genes, and proteins and exhibit stimulus responsiveness making them “smart” nanocarriers [77,78]. Liposomes are limited by their poor stability and possibility of triggering an immune response, while micelles are limited by their occasional cytotoxicity and degradability [60]. Several triggering mechanisms can be used to stimulate the release of encapsulated cargo from the nanocarriers [79]. The different types of nanocarriers and possible release trigger mechanisms are presented in Figure 2.

A detailed discussion on GNP inorganic hybrids for cancer therapy is beyond the scope of this paper and can be a topic of a separate extensive review. The next subsections focus on organic GNP nanohybrids, namely, liposome-based and polymer-based GNP hybrids.

## 3. Organic GNP Nanohybrid Chemotherapeutic Platforms

### 3.1. Multimodal Liposome–GNP Nanohybrids

The temperature responsiveness of some nanocarriers, such as liposomes and polymers, makes them suitable vehicles to be hybridized with heat-generating nanomaterials, such as GNPs [48,49]. Several studies explored hybridizing GNPs with thermosensitive nanocarriers to achieve combined hyperthermia-triggered drug release and the thermal ablation of tumor cells [34,38,39,80,81,82,83]. Likewise, some nanocarriers can respond to internal stimuli such as the TME acidic pH [38,39,81], thereby allowing the utilization of multiple stimuli to trigger drug release. One of the materials investigated for GNP hybridization due to their heat responsiveness is liposomes [34,38,39,80,81,82,83]. Liposomes [84] have greatly impacted drug delivery applications by improving the stability, cellular uptake, biodistribution, and biocompatibility of several drugs. Since the first focus on their clinical potential in the 1980s, liposomes have been used to encapsulate hydrophilic and hydrophobic drugs, nucleic acids, proteins, and imaging probes. Advances in liposome-mediated drug delivery were covered by Sercombe et al. [85] and O. B. Olusanya [86]. Low-temperature-sensitive liposomes (LTSLs) capable of undergoing phase transition at low temperatures serve as ideal temperature-responsive carriers due to their ability to respond to mild hyperthermia, which is harmless to normal tissues [80]. LTSLs are used to deliver drugs via mild hyperthermia, such as phase III FDA-approved ThermoDox^®^, which uses LSTLs to deliver DOX [87]. Despite their numerous advantages, liposomes still suffer from some drawbacks, including their poor drug release and low retention time at the tumor site, which reduce the efficacy of the treatment [88]. GNP–liposome nanohybrids could improve drug release and, thus, therapeutic efficacy. 

Koga et al. studied liposome–GNP nanohybrids for the delivery of chemotherapeutic drug doxorubicin (DOX) as a potential strategy to overcome limitations associated with FDA-approved nanoformulation Doxil^®^ (PEGylated liposomal DOX) [34]. Doxil^®^’s prolonged circulation time due to the presence of PEG is known to cause palmar–plantar erythrodysesthesia, an adverse dermatological skin reaction caused by certain chemotherapeutic drugs [89]. Furthermore, Doxil^®^ was found to utilize the endocytic pathway to enter the cell, which leads to the lysosomal sequestration of the nanocomposite, which could prevent DOX from entering into the nucleus [90], its main site of cytotoxic action [91]. To overcome those limitations, Koga et al. covalently coated thermosensitive PEGylated liposomal DOX with a surface gold nanoshell to achieve a temperature-triggered release of DOX. This study reported the effective gold-nanoshell conversion of NIR light to heat, the induction of heat-induced liposomal phase transition and subsequent DOX release, the biocompatibility of the nanocomposite, and a significantly enhanced eradication of tumor cells via synergistic DOX/hyperthermia effects compared with single DOX or single hyperthermia treatments in vitro. Although the work by Koga et al. claimed to improve the bioavailability of DOX using the GNP-coated thermoresponsive liposomes, the researchers failed to describe the mechanism by which incorporating GNP into Doxil^®^ could avoid lysosomal sequestration [34]. GNP/DOX-loaded liposomes could possibly evade lysosomal entrapment by (1) rupturing the lysosome upon photothermal conversion or (2) causing the heat-triggered release of DOX outside of the cancer cells, thereby making DOX available for all cancer cells at that site. Synergistic PTT/chemotherapy delivery using thermosensitive liposomal GNPs was also studied by Xing et al. [38]. Interestingly, this work utilized two stimuli, heat and low TME-characteristic pH, to trigger DOX release. Xing et al. reported high NIR-to-heat conversion efficiency and successful DOX release via dual heat-induced liposomal phase transition and low-pH-induced membrane instability. Importantly, the GNP–liposome nanohybrid exhibited superior cytotoxicity in vitro and in vivo due to the synergistic PTT–chemotherapy activity while causing negligible systematic toxicity in vivo [38]. 

Another work by Thakur et al. [92] exploited GNP-incorporated thermosensitive liposomes but delivered a photosensitizer rather than a drug to achieve combined photodynamic therapy (PDT) and PTT. In addition to combining PDT and PTT, this strategy could overcome the limitation of hydrophobicity associated with fluorescent PDT photosensitizer zinc phthalocyanine (ZnPc) by shielding it within liposomes. The GNP-encapsulated ZnPc liposomes showed the efficient entrapment of ZnPc, stability under storage and physiologic conditions, and effective photothermal conversion ability that efficiently triggered ZnPc release. In addition, the nanohybrid retained ZnPc-characteristic fluorescence, efficiently generated singlet oxygen for PDT, and significantly improved internalization and cancer cell growth inhibition in vitro, which substantially inhibited tumor growth due to PDT/PTT synergism [92]. Although this nanohybrid was not used to deliver drugs, it still has the potential to carry and deliver anti-cancer drugs with ZnPc, thereby combining the cytotoxic effects of the delivered drug, PDT, and PTT in a single composite. In addition to tumor annihilation, the fluorescent properties of this nanohybrid could make possible its future utilization for diagnosis or image-guided multimodal delivery/PDT/PTT. 

Furthermore, gold nanomaterials were reported to improve PDT by several papers [93,94,95], further extending their potential for PDT therapy and combination with other approaches, such as PTT. Kautzka et al. delivered both a photosensitizer (Rose Bengal) and a chemotherapeutic drug (DOX) using NIR light stimulus for dual enhanced PDT and chemotherapy toxicity. This work reported an improved GNP-induced generation of singlet oxygen species and PDT/chemotherapy cell death in vitro. However, the maximum cell death reported did not exceed 38%. This could have been due to the insufficient heat generated to induce liposomal phase transition (45 °C) at the chosen NIR wavelength [96]. Ou et al. [80] co-delivered LSTL-encapsulated DOX and multi-branched gold nanoantennas (MGNs) for combined heat-triggered DOX delivery and PTT in triple-negative breast cancer in vitro. The co-delivered MGNs and DOX-LSTLs achieved efficient cellular internalization and induced significant cell death in vitro due to NIR-induced heat generation from MGNs and resulting DOX release from LSTLs. Therefore, this study achieved a light-activated, controlled drug delivery that could evade the typical DOX-associated off-target toxicities [80]. 

Another study by Won et al. improved liposome–GNP nanohybrid drug delivery within a chitosan hydrogel as a reservoir to retain the nanocomposite in the TME [88]. Chitosan was used as a reservoir system due to its ability to undergo a solid–gel phase transition in response to temperature. Importantly, chitosan is a biocompatible and biodegradable polymer with low toxicity and immune response. The researchers reported significant improvement in nanohybrid localization and retention at the tumor site, efficient and sustained heat generation in response to NIR with subsequent DOX release, and significant inhibition of tumor growth while maintaining a good systematic safety profile [88]. In a similar work, Wang et al. utilized chitosan-modified liposomes coated with a gold nanoshell for combined PTT and dual pH/temperature resveratrol (anti-cancer drug) release. The results showed efficient heat generation by the gold nanoshell surpassing that reported for gold nanostars or nanorods and enhanced pH responsiveness due to the presence of amine groups on chitosan. Moreover, increased temperature responsiveness due to the presence of the thermosensitive liposomes was observed, supported by the enhanced resveratrol release in response to the dual pH/temperature stimuli. In vitro analyses showed efficient cellular uptake enhanced by NIR and improved cell death due to resveratrol and PTT synergy [39]. Similarly, Luo et al. utilized GNP–liposome nanohybrids with chitosan for dual pH/temperature oleanolic acid (anti-cancer drug) release. The study reported efficient low-pH- and heat-triggered oleanolic acid release and enhanced chemo-photothermal killing of cancerous cells compared with single chemotherapy or photothermal therapy in vitro and in vivo [81]. 

Unlike most studies that conjugate GNPs to the liposomal surface, He et al. encapsulated DOX-loaded gold nanocages within thermosensitive liposomes. The liposomal coating was used to improve the stability and biocompatibility of the GNPs. The study showed that coating the GNPs with liposomes and loading DOX did not influence the gold nanocages’ photothermal properties but increased their cellular uptake and nuclear localization. The conversion of NIR light to heat efficiently triggered DOX release due to the liposomes’ phase transition and induced significant tumor cell eradication via hyperthermia/DOX synergy in vitro [82]. In a study by Singh et al., nanogold-coated liposomes were similarly used to load the anti-cancer drug curcumin [97]. The study reported high curcumin loading efficiency, efficient conversion of NIR light to heat, dual PTT- and hyperthermia-triggered curcumin release, significant enhancement in cellular uptake, and in vitro PTT-/curcumin-induced cell death [97]. Several other similar studies utilized liposome–GNP nanohybrids for the delivery of different drugs to improve their bioavailability (e.g., poorly water-soluble betulinic acid), avoid systematic side effects (e.g., DOX), and essentially achieve enhanced tumor annihilation [81,98,99,100,101,102,103]. Table 1 presents a summary of these studies. 

Another study by Li et al. utilized immune-targeted GNP-coated liposomes modified with a HER-2 antibody to deliver the drug cyclopamine, a drug capable of stroma destruction and tumor cell eradication [104]. The proposed nanoformulation was reported to induce significant toxicity against tumor cells in vitro and in vivo, due to combined chemotherapy/PTT and deep tumor penetration compared with single chemotherapy or PTT treatments. Additionally, HER2 surface modification increased the cellular uptake of the drug-loaded nanocomposite in vitro and in vivo. Notably, the nanocomposite maintained a good safety profile in vivo [104]. 

Another strategy explored by Zhang et al. used ammonium bicarbonate (ABC)- and DOX-encapsulated liposomal gold nanorods (GNRs) for image-guided, NIR-triggered drug release. Upon exposure to NIR light, ABC decomposes and generates carbon dioxide, causing transient cavitation that can promote DOX release. The DOX/ABC-loaded liposomal GNRs were also decorated with folic acid to achieve tumor targeting. Furthermore, GNRs were also used as CT contrast agents to achieve image-guided chemotherapy delivery to tumor cells. In vitro and in vivo studies served as good CT contrast agents and showed increased tumor inhibition upon NIR exposure compared with ABC-lacking composites [105]. On the other hand, Rengan et al. developed thermosensitive GNP-modified liposomes for hyperthermia-triggered drug release, PTT, and CT imaging. The results showed efficient PTT and PTT-induced cell death in vitro, the heat-triggered release of the model drug/dye calcein, and CT contrast of the GNP liposomes [106].

In addition to solid GNPs, thermoresponsive liposomes were also studied with hollow GNPs (HGNPs). HGNPs gained attention over solid GNPs, particularly for drug delivery purposes, due to the presence of an inner cavity capable of drug hosting and possessing higher photothermal conversion abilities [83]. Similar to solid GNPs, HGNPs come in different morphologies, such as spheres, rods, stars, and cages. Their use for biomedical applications was reviewed by Park et al. [107]. Several studies explored bare HGNPs to encapsulate drugs and achieve heat-triggered drug release with/without PTT, as reported by You et al. [108] and Xiong et al. [109]. Those studies incorporated groups that can be cleaved via heat generation, such as surface peptides linked to GNPs through Au-S bonds [109]. A study that compared solid GNP–liposome nanohybrids with hollow GNP–liposome nanohybrids reported an eight-fold enhancement of anticancer activity from chemotherapy–hyperthermia coaction using hollow GNP-loaded liposomes [83].

Other studies explored liposome–GNP nanohybrids as drug carriers without the use of triggering stimuli or stimuli other than temperature [43,110,111,112,113,114]. Sonkar et al. [110] reported the use of transferrin-coated liposomes encapsulating chemotherapy docetaxel and GNPs. This transferrin-targeted nanoformulation achieved sustained docetaxel release, a higher tumor cell eradication at a lower concentration compared with the marketed docetaxel, and higher cellular uptake compared with their non-targeted counterparts. Although this work did not benefit from photothermal conversion, this nanoformulation could be further modified by utilizing thermoresponsive delivery and dual chemotherapy/PTT actions for multimodal therapy [110]. Hamzawy et al. delivered the drug temozolomide via intratracheal inhalation using GNP–liposome hybrids as nanocarriers. The nanocomposite showed improved in vivo drug delivery while avoiding systematic toxicity [113]. Another study by Zhang et al. delivered PTX from GNP–liposome nanohybrids via diffusion, glutathione (GSH)-induced release, and enzyme-mediated release [112]. GSH is a commonly upregulated antioxidant in cancers to counteract oxidative stresses [115]. Therefore, GSH provides a tumor-specific endogenous stimulus for drug delivery purposes [116]. Bao et al. [43] also used GNP–liposome hybrids to deliver chemotherapeutic drug paclitaxel using the enzyme esterase and the antioxidant GSH as triggers. This study reported sustained intracellular paclitaxel release, improved blood circulation time, and enhanced anti-cancer activity in vivo [43]. Furthermore, liposomal GNPs were also used to deliver genes in addition to drugs without utilizing GNPs for heat-related effects (PTT or heat-triggered release) or radiotherapy [114].

GNP–liposome nanohybrids were also explored for single PTT or hyperthermia-triggered drug release. PEG-coated liposomal GNPs were studied for single PTT and were found to exhibit enhanced PTT, cell cytotoxicity, and passive targeting abilities in vivo [117]. Likewise, Kwon et al. released DOX from GNP–liposome hybrid nanostructures using NIR-generated heat and reported efficient DOX encapsulation and NIR-triggered release compared with GNP-negative thermosensitive liposomes. As a result, the nanocomposite induced tumor growth inhibition in vitro and in vivo upon DOX loading and NIR exposure [118].

Other liposome–GNP nanohybrids were used for the triggered delivery of proteins and genes, as reported by Du et al. [119], Refaat et al. [120], and Grafals-Ruiz [121]. Gene therapy is one of the promising strategies explored for cancer treatment in which genes are either: (1) provided to translate to a disease-curing protein [119] or (2) delivered to cells to regulate the expression of certain genes [119,122]. RNA interference is a commonly used type of gene therapy that involves the use of an RNA molecule to knock down a target gene. Small interfering RNA (siRNA) was studied for such inhibition of genes by targeting messenger RNAs. Although promising, treatment via siRNA is greatly limited by RNA instability and susceptibility to degradation. 

Jia et al. [122] used liposomal GNPs to deliver siRNA to the mutant oncogene K-Ras in vitro and in vivo for dual siRNA and PTT tumor eradication. A photothermal nanomaterial, Prussian blue analog (PBA), gold nanoflowers, targeting RGD peptides, and liposomes were incorporated into a single composite to achieve dual NIR-triggered siRNA release and PTT (gene therapy–PTT synergy). This composite could achieve gene therapy–PTT coaction guided by three imaging modalities: CT imaging, photoacoustic imaging (PAI), and photothermal imaging (PTI). Owing to the synergism between the components of the nanohybrid, it achieved increased accumulation at the tumor site, significant siRNA-induced inhibition of K-Ras expression, and significant inhibition of tumor cell growth in vitro and in vivo upon NIR exposure. In terms of imaging abilities, the nanohybrid improved PAI, PTI, and CT imaging, thereby indicating the composites’ potential for image-guided therapy [122]. Liposomal GNPs were also used to deliver interfering RNAs (RNAi) across highly selective biological barriers, such as the BBB. Grafals-Ruiz et al. used RNAi-functionalized GNPs entrapped within liposomes and targeted via BBB-targeting peptides for glioblastoma treatment. This study reported efficient cellular internalization and the inhibition of the overexpressed microRNA (miRNA-92b) involved in glioblastoma growth and progression, both in vitro and in vivo. However, this study did not benefit from any triggering stimulus to control the release of RNAi [121]. Likewise, liposomal GNPs were exploited for the delivery of both nucleotides and drugs. Skalickova et al. encapsulated fluorescent drugs (DOX, ellipticine, and/or etoposide) and the antisense oligonucleotide that can block the N-myc protooncogene. The formulations demonstrated the suitability of the liposomal gold nanoparticles for delivering both drugs and oligonucleotides. However, this study did not employ any triggering mechanism and did not assess the biocompatibility or the tumor-killing ability of the nanohybrid in vitro or in vivo [123]. 

Other studies utilized GNP–liposome nanohybrids but did not benefit from the GNP photothermal properties for triggered drug release or thermal ablation. However, the incorporation of GNPs into those nanocomposites suggests their possible future utilization for photothermal conversion. Liposome-coated GNP nanohybrids loaded with the antimitotic drug docetaxel (DTX) were studied by Kang et al. The results showed efficient entrapment of DTX within the lipid bilayer, controlled untriggered DTX release, increased cellular uptake and significant toxicity surpassing that of the free drug in vitro [124]. Another study by Kunjiappan et al. also exploited liposome–GNP nanohybrids to deliver epirubicin, a chemotherapeutic agent targeting lymph-node-metastasized breast cancer, and reported similar satisfactory results [125]. Table 1 summarizes GNP–liposome nanohybrids for triggered drug delivery purposes. It is worth noting that most GNP–liposome thermosensitive nanohybrids target breast cancer, the most commonly diagnosed cancer, as of 2020 [126]. Based on 2020 cancer statistics, female breast cancer was responsible for the most cancer-caused mortalities in twelve regions around the world, surpassing lung cancer [127]. Despite the improved life expectancy, 30% of breast cancer patients inevitably progress to the metastatic, incurable form of the disease [126,128]. Those statistics only indicate the need for improved breast cancer treatment strategies.

In addition to their photothermal properties, liposomal GNPs are exploited for imaging purposes such as CT contrast probes [129]. Hence, they can be potentially combined with other applications, such as diagnosis, image-guided drug delivery, and PTT features.

**Table 1:** Multimodal GNP–liposome nanohybrids for cancer therapy.

Triggering Stimuli	Loaded Agents and Surface Modifications	Targeted Cancer Type	Release Mechanisms	Toxicity	References
NIR-generated heat	DOX PEG	Lung cancer	DOX release via heat-induced liposomal phase transition Hyperthermia and DOX synergy	PTT/chemotherapy toxicity in vitro	[34]
NIR-generated heat Low pH	DOX	Cervical cancer	DOX release via heat-induced liposomal phase transition and low-pH-induced membrane instability Hyperthermia and DOX synergy	PTT/chemotherapy toxicity in vitro and in vivo	[38]
NIR-generated heat	ZnPc	Breast cancer	Heat-triggered ZnPc release PDT and PTT synergy	PTT/PDT toxicity in vitro and in vivo	[92]
NIR-generated heat	DOX	Breast cancer	DOX release via heat-induced liposomal phase transition Hyperthermia and DOX synergy	PTT/chemotherapy toxicity in vitro and in vivo	[83]
NIR-generated heat	DOX	Breast cancer	DOX release via heat-induced liposomal phase transition Hyperthermia and DOX synergy	PTT/chemotherapy toxicity in vitro	[80]
NIR-generated heat	DOX Chitosan	Melanoma	DOX release via heat-induced liposomal phase transition	Chemotherapy-induced toxicity in vitro and in vivo	[88]
NIR-generated heat Low pH	Resveratrol Chitosan	Cervical cancer	Resveratrol release via pH-induced chitosan amine group protonation and heat-induced liposomal phase transition Hyperthermia and resveratrol synergy	PTT/resveratrol toxicity in vitro	[39]
NIR-generated heat Low pH	Oleanolic acid Chitosan	Osteosarcoma	Dual pH- and temperature-stimulated oleanolic acid release Hyperthermia and oleanolic acid synergy	PTT/oleanolic acid toxicity in vitro and in vivo	[81]
NIR-generated heat	DOX	Breast cancer	DOX release via heat-induced liposomal phase transition Hyperthermia and DOX synergy	PTT/chemotherapy toxicity in vitro and in vivo	[82]
NIR-generated heat	DOX	Liver cancer Breast cancer	DOX release via heat-induced liposomal phase transition Hyperthermia and DOX synergy	PTT/chemotherapy toxicity in vitro	[99,103]
NIR-generated heat	Betulinic acid	Cervical cancer Osteosarcoma	Betulinic acid release via heat-induced liposomal phase transition Hyperthermia and betulinic acid synergy	PTT/betulinic acid toxicity in vitro and in vivo	[98,100]
NIR-generated heat	Curcumin	Melanoma	Curcumin release via heat-induced liposomal phase transition Hyperthermia and curcumin synergy	PTT/chemotherapy toxicity in vitro	[97]
NIR-generated heat	DOX PEG Low-density lipoprotein receptor (LDLR)-binding peptide	Prostate cancer	LDLR-binding-peptide-mediated cellular uptake and tumor accumulation. DOX release via heat-induced liposomal phase transition Hyperthermia and DOX synergy	PTT/chemotherapy toxicity in vitro and in vivo	[101]
NIR-generated heat	DOX Folic acid	Breast cancer	Folic acid-mediated cellular uptake and tumor accumulation DOX release via heat-induced liposomal phase transition Hyperthermia and DOX synergy	PTT/chemotherapy toxicity in vitro and in vivo	[102]
NIR-generated heat	HER2 Cyclopamine	Breast cancer	Deeper tissue penetration via cyclopamine stroma destruction HER2-mediated tumor targeting Cyclopamine release via heat-induced liposomal phase transition Hyperthermia and cyclopamine synergy	PTT/chemotherapy toxicity in vitro and in vivo	[104]
NIR-generated heat	DOX ABC Folic acid	Breast cancer Sarcoma (S180) ascite cells were used for in vivo studies	DOX release via transient cavitation caused by carbon dioxide generated upon hyperthermia-induced ABC decomposition Improved tumor cell targeting via folic acid-mediated endocytosis Computed tomography contrast agent	Chemotherapy-induced tumor inhibition in vitro and in vivo	[105]
NIR-generated heat	PEG	Breast cancer	Tumor eradication via NIR-generated PTT	PTT-induced tumor growth inhibition in vitro and in vivo	[117]
NIR-generated heat	DOX	Breast cancer	DOX release via heat-induced liposomal phase transition	Chemotherapy-induced tumor inhibition in vitro and in vivo	[118]
NIR-generated heat	Calcein	Breast cancer	Hyperthermia-triggered calcein release Tumor eradication via NIR-generated PTT	PTT-induced cell death in vitro	[106]
NIR-generated heat	DOX Rose Bengal	Colon cancer Breast cancer	GNP-induced generation of singlet oxygen species (PDT) and DOX release PDT and DOX synergy	PDT and DOX-induced toxicity in vitro	[96]
NIR-generated heat	siRNA PBA RGD	Pancreatic cancer	PBA/GNP NIR-triggered siRNA release and PTT Gene therapy–PTT synergy PAI, PTI, and CT imaging contrast agents	K-Ras knockdown and PTT-induced toxicity in vitro and in vivo	[122]
NIR	DOX	Breast cancer	NIR-triggered DOX release	DOX-induced toxicity in vitro	[130]
Low pH Heat	DOX	Ovarian cancer Breast cancer	Low-pH- and hyperthermia-triggered DOX release	DOX-induced toxicity in vitro (to a lower extent than free DOX)	[131]

### 3.2. Multimodal Polymer–GNP Nanohybrids

Polymeric nanocarriers are another group of smart nanovehicles that can improve the performance of traditional cancer therapeutics [132,133]. These polymeric drug delivery systems can be further modified to induce stimulus responsiveness and improve their performance [133]. Chitosan alginates and deoxyribonucleic acid are interesting natural polymeric nanocarriers for drug delivery purposes due to their natural biocompatibility, stimulus responsiveness, and hydrophobic/hydrophilic drug encapsulation [134,135]. For instance, one of the materials that polymers were hybridized with is GNPs. Zhang et al. functionalized the GNP surface with DNA and an affibody (HER2-specific antibody mimetic) to provide HER2 targeting to tumor cells for 5-fluorouracil and DOX co-delivery. Interestingly, this work reported the effective loading of both drugs and acidic pH- and DNase II (nuclease)-triggered drug release [136]. Low pH and high DNase II expression levels are both tumor-specific features that can ensure drug release specifically at the tumor site [37,137]. Furthermore, the internalization rate of the drug-loaded GNP nanohybrids of HER2-overexpressing cancer cells increased due to affibody-receptor-mediated endocytosis in vitro. In vitro studies also showed the biocompatibility of nanohybrids and improved cytotoxic effects surpassing those of the free drug combination in HER2-overexpressing breast cancer cells due to DOX/5–fluorouracil synergy and affibody-mediated internalization [136].

Poly(lactic-co-glycolic acid) (PLGA), another smart polymeric nanocarrier, was explored as a thermosensitive drug carrier for combined heat-induced drug delivery and PTT. Park et al. utilized DOX-loaded PLGA, half-coated with GNPs, for dual chemotherapy delivery and PTT. The formulation exhibited high biocompatibility, enhanced cytotoxicity compared with single DOX or single PTT treatments due to DOX/PTT synergy, and the effective internalization of the nanohybrid in vitro [138]. Another polymeric GNP hybrid investigated by Adeli et al. used polyrotaxanes to shelter GNPs for heat-triggered DOX and cisplatin release. Polyrotaxanes are highly functional and biocompatible assemblies of α-cylodextrin rings supramolecularly anchored to PEG axes that can improve the internalization rate of nanocomposites of tumor cells. Light-to-heat conversion by GNPs induced polyrotaxane shell cleavage leading to the effective release of the encapsulated drugs and induced cytotoxicity comparable to that of free drugs while maintaining compatibility in vitro. However, even though the nanohybrid successfully induced the death of cancer cells, the viability of the cells was not reduced below ~40% for DOX and ~50% for cisplatin, respectively [33]. The GNP/polyrotaxane nanohybrid’s cytotoxic effects could be intensified by combining the heat-induced drug release with photothermal therapy, radiotherapy, or maybe both. 

Other GNP polymeric nanohybrids were studied without utilizing the GNP photothermal properties for triggered drug release, thermal ablation, or radiotherapy. Dai et al. [139] hybridized GNPs with protein polymers to endue the nanocomposite with biocompatibility and improve the uptake of the hydrophobic drug curcumin. A significant enhancement in the GNP/protein polymer binding and the in vitro cellular uptake of the drug curcumin were observed. Moreover, curcumin exhibited a sustained release profile compared with GNP-free protein polymers. However, this study did not assess the toxicity of this system against cancer cells [139]. Future improvements in this hybrid nanostructure could be obtained by utilizing other GNP features, such as photothermal conversion effects. In the following subsections, some of the most common polymers hybridized with GNPs for cancer therapy, hydrogels and micelles, are discussed. 

#### 3.2.1. Multimodal Hydrogel–GNP Nanohybrids

Hydrogels represent physically or covalently crosslinked, natural, synthetic, or semi-synthetic hydrophilic polymer networks [132]. Hydrogels are among the promising polymeric nanocarriers utilized for drug delivery due to a range of desirable properties, including (i) their biocompatibility due to their high water content; (ii) porosity, which allows the encapsulation and delivery of drugs to be performed; (iii) controlled drug release via hydrogel swelling/shrinkage; (iv) soft deformable nature [140,141]; and (v) biodegradability [64,142]. In addition, nanohydrogels (sizes typically between 20 and 250 nm) can cross biological barriers and provide intracellular access for cargo delivery [63]. Yet, hydrogels possess some limitations, including their inability to host hydrophobic drugs; the rapid release of encapsulated drugs due to large pore size and high water content; and deformability, which could be insufficient for injectable formulations [141]. Although much progress has been achieved with hydrogels in the biomedical field, their performance could be further improved if their limitations were circumvented. Hydrogels/nanogels were hybridized with other nanomaterials such as magnetic nanoparticles [36,143,144,145], GNPs [146,147,148], and metal-organic frameworks (MOFs) [149]. Hybridization can impart additional properties, such as multifunctionality [149] and/or specific stimulus responsiveness [52,150,151] to the gel system, thereby improving their performance.

Hybridizing hydrogels with GNPs was explored as one of the strategies to achieve more efficient cancer treatment [152]. As with liposomes, hydrogels can also be thermoresponsive and be used for specific heat-triggered drug release. Such thermoresponsive gels undergo a sol-to-gel transition when heated to a specific temperature (i.e., low critical gelation temperature), leading to drug release [153,154]. Several studies investigated thermo- and non-thermoresponsive GNP–hydrogel hybrids for cancer therapy. In those studies, GNPs–hydrogels were mostly utilized for synergistic cancer eradication, such as dual PTT and chemotherapy [155] or triple PTT, chemotherapy, and radiotherapy [24]. 

Alginate is a commonly used hydrogel polymer for biomedical applications due to its high biocompatibility, easy gelation, low toxicity, and relatively low price [156]. Several studies investigated GNP–alginate hydrogels for cancer therapy, particularly enhancing radiotherapy/chemotherapy delivery, and combined approaches such as dual chemotherapy and PTT or triple chemotherapy, radiotherapy, and PTT [24,30,157,158]. In the study by Alamzadeh et al., an alginate hydrogel was loaded with cisplatin and GNPs, where the GNPs were used for PTT and radiosensitization. The results showed significantly reduced apoptotic cell death in response to the tri-modal therapy compared with the single or dual synergistic treatments, with negligible in vitro toxicity [24]. However, this study exploited heat as an adjuvant to chemotherapy and radiotherapy without using it as a trigger for cisplatin release. Therefore, future improvements in this nanohybrid include using a thermoresponsive polymer with or without alginate and utilizing GNP photothermal conversion to trigger chemotherapy release. This was conducted by Mirrahimi et al., who used the same alginate/GNP/cisplatin composite for local triple synergistic therapy but utilized heat as a drug delivery trigger. This work conducted in vivo studies to assess the formulation’s hematological effects. The study reported heat-triggered cisplatin release via hydrogel degradation in vitro, photothermal conversion ability, and the highest apoptotic anti-tumor performance compared with bi- or unimodal therapies while maintaining a good safety profile in vivo [157]. The same nanocomposite was also explored for dual chemotherapy/PTT, where it achieved enhanced cell death compared with single chemotherapy or PTT in vitro [158]. Likewise, for dual chemotherapy/radiotherapy [30], the nanocomposite induced significant tumor growth inhibition via apoptotic cell death while maintaining biocompatibility in vivo [30]. However, neither study took advantage of heat-responsive cisplatin delivery, thereby leaving room for further improvement in the nanocomposite by incorporating heat responsiveness. GNP/alginate/cisplatin nanohybrids were also studied by Keshavarz et al. for computed tomography (CT)-image-guided drug delivery. The nanocomposite achieved higher toxicity in vitro than free cisplatin and enhanced CT imaging in vitro. However, this study did not utilize the GNP photothermal conversion abilities to kill cancer cells or responsively trigger drug release [25].

Alginate-based thermosensitive hydrogels with GNPs were also studied for cancer therapy by Kiseleva et al., who used alginate combined with a thermosensitive polymer, PF127, to make thermoresponsive hydrogels for the encapsulation and release of GNPs. In this study, GNPs were used as the therapeutic agent to be released from the gel via gel dissolution and GNP diffusion without being triggered by heat [153]. Another alginate-based nanohybrid hosting both GNPs and iron oxide nanoparticles was studied for magnetically targeted drug delivery, PTT, and magnetic resonance imaging (MRI). The iron oxide nanoparticles provided the nanocomposite with magnetic responsivity, thereby allowing the magnetic targeting of the nanohybrid to the tumor site to be performed. In addition, they enhanced MRI by acting as a T2 contrast agent. In this study, DOX-loaded GNP/MNP/alginate composites enhanced magnetic resonance imaging and significantly induced tumor inhibition in vivo via PTT/chemotherapy synergy, which was further enhanced by magnetic guidance, while maintaining low toxicity in vivo [159].

Chitosan is another interesting natural polymer used in biomedicine for its biocompatibility, low toxicity, biodegradability, low immunogenicity, and temperature-induced sol-to-gel transition. Won et al. used chitosan hydrogels to hold GNP–liposome DOX, thereby providing an injectable hydrogel nanohybrid that served as a reservoir of the liposomal DOX and responsively released DOX upon NIR exposure. Significant tumor reduction in vivo due to DOX release via NIR-generated hyperthermia while avoiding systematic toxicity was reported [88]. GNP–chitosan nanogels were also explored for dual drug release and PTT. Thermoresponsive nanogels were synthesized by grafting PNIPAAm onto chitosan and incorporating GNPs to achieve dual-triggered curcumin release and PTT. In this study, curcumin achieved an efficient low-pH- and hyperthermia-triggered release, biocompatibility, efficient nanohybrid endocytic internalization by cancer cells compared with normal cells, and curcumin-/PTT-induced toxicity in vitro [35]. Xia et al. used heat-responsive chitosan to hold GNPs hosted within a porous silica nanoparticle (PSiNP) matrix for triggered chemotherapy release and PTT. This study aimed at providing a nanohybrid that could serve as a long-term PTT agent to avoid repeated treatment injections. Chitosan/PSiNPs/GNPs carrying DOX achieved low-pH- and NIR-responsive release and significantly inhibited tumor growth in vivo while having a good safety profile. Importantly, the chitosan encapsulated PSiNPs/GNPs maintained a longer, more persistent photothermal conversion in contrast to the uncoated PSiNPs/GNPs, which degraded in the absence of chitosan protection [160]. 

**Figure 3:**
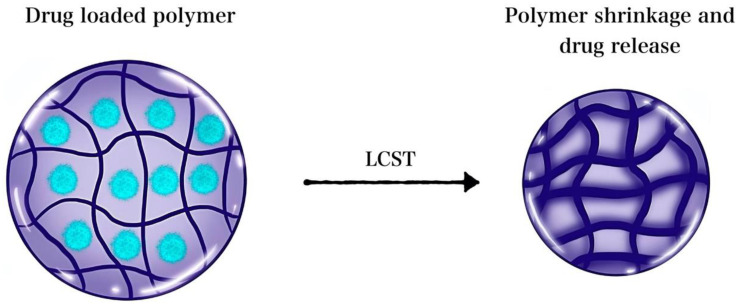
A schematic illustrating the ‘coil-to-globule’ phase transition of thermosensitive polymers upon exposure to heating above the LCST. The conformation change from the hydrated coil state (**left**) to the dehydrate globule state (**right**) results in drug release.

Poly(N-isopropylacrylamide) (PNIPAAm), a polyalkylacrylamide derivative, is another thermoresponsive polymer that was used for the fabrication of heat-responsive polymeric nanosystems, including hydrogels. PNIPAAm has a low critical solution temperature (LCST) of 32 °C. The critical temperature entails the behavior and conformational change of the polymer upon exposure to cooling or heating. Typically, when the temperature is raised above the polymer LCST, the polymer chains undergo a reversible volume phase transition. Initially, the polymers exist in a homogenous hydrated state, where the load is retained; however, once heated, they deform and release their contents, as illustrated in Figure 3 [147,161,162]. Furthermore, the PNIPAAm LCST can be tailored via copolymerization to trigger release at temperatures higher than the body temperature, thereby ablating tumor cells while simultaneously triggering the release of loaded cargo via polymer volume phase transition.

Pourjavadi et al. studied GNP hybrid nanogels for heat-triggered DOX release. They utilized PNIPAAm and carboxymethyl chitosan polymers to fabricate heat-sensitive nanogels, hybridized with GNPs and magnetic iron oxide nanoparticles. The hybrid nanogel resulted in controllable temperature-induced DOX release, significant toxicity, and biocompatibility in vitro. However, those results could be improved using magnetic targeting [36]. Ghorbani et al. used GNP–PNIPAAm-based nanogels for the delivery of the chemotherapeutic drugs DOX and 6-marcaptopurine. The nanogel/GNP hybrid contained PNIPAAm combined with a pH-responsive polymer (maleic acid) and a redox-responsive polymer (N,N’-bis(acryloyl)cystamine), thereby achieving drug release control at three levels: temperature, redox, and pH. This study reported rapid tumor-specific pH-, redox-, and temperature-triggered drug release, hemocompatibility, and similar or improved cytotoxicity compared with single or combined DOX/6-marcaptopurine. However, although this study showed the temperature responsiveness of the GNP hybrid nanogel, the heat used was not generated by the GNPs themselves. Furthermore, the ability of the GNP hybrid nanogel to undergo photothermal conversion for effective heat generation was not assessed. It is always necessary to test whether the GNP photothermal conversion feature is still retained after hybridization or not [147]. Hence, even though this work provided important information, it is important to prove that the GNP nanogel can generate heat and that this heat is sufficient to trigger drug release from the nanogel. 

As with liposomes, HGNPs were also hybridized with temperature-responsive polymeric nanocarriers for temperature-triggered release. Solorzano et al. used HGNP-decorated PNIPAAm nanogels to carry and release the drug bupivacaine via NIR-to-heat conversion. The results showed a rapid increase in temperature upon photothermal conversion leading to the shrinkage of the GNP hybrid nanogel and the expulsion of the loaded drug. Moreover, in vitro biocompatibility within a certain concentration limit was reported [163]. Yavuz et al. coated the surface of hollow gold nanocages with the copolymer PNIPAAm-co-polyacrylamide via Au-S bonds to achieve the triggered release of the loaded effector molecules. The polymer’s closed pores trapped the loaded molecules until a temperature of 39 °C was reached, causing the polymer’s pores to open up and release the loaded molecules [164]. As with the above-described studies, this work combined PNIPAAm with polyacrylamide to tailor the polymer LCST for temperatures above the body temperature. Yavuz et al. also loaded polymer-coated hollow gold nanocages with DOX to test the nanohybrid’s thermoresponsive properties and reported the controlled release of DOX upon exposure to NIR laser and the DOX-induced killing of breast cancer cells in vitro [164]. 

Several other GNP hydrogel nanohybrids composed of different polymers were studied for cancer therapy by achieving dual chemotherapy/PTT [155,165,166,167,168,169], chemoradiotherapy [170], single PTT [171], and others [172]. Table 2 summarizes some of these studies. Li et al. reported chemotherapy-resistance reversal in vitro using GNP nanogels. They used GNP–hyaluronic acid nanohydrogels to carry DOX and release it via dual hyperthermia/GSH stimuli, while actively targeting tumor cells via hyaluronic acid–CD44 receptor interactions. This nanogel/GNP hybrid responsively released DOX in response to heat and GSH, actively endocytosed into cells via hyaluronic acid/CD44 binding, and achieved reversal of chemotherapy resistance in vitro [173]. It is important to note that such resistance, particularly multidrug resistance, is responsible for more than 90% of cancer mortalities in chemotherapy-treated patients [174].

#### 3.2.2. Multimodal Micelle–GNP Nanohybrids

Another family of smart polymeric nanocarriers exploited for stimulus-assisted drug delivery for cancer treatment is micelles. Micelles are spherical aggregates of amphiphilic, self-assembling building blocks with sizes typically ranging between 10 and 100 nm [75,76]. Micelles possess hydrophobic cores and hydrophilic shells, deeming them suitable for the encapsulation and solubilization of hydrophobic drugs [77]. They were explored as GNP-incorporated temperature-responsive carriers for dual chemotherapy delivery and PTT. Sun et al. investigated the use of pluronic-poly(L-lysine) (PLL) micelles coated with GNPs and loaded with paclitaxel for bimodal chemotherapy/PTT. The study reported the temperature-responsiveness of the GNP-coated micelles, heat-triggered paclitaxel release, hemocompatibility, cytocompatibility, enhanced therapeutic effect, and increased cellular uptake in vitro. Furthermore, the GNP–micelle hybrid improved targeting and cytotoxicity while maintaining biosafety in vivo [175]. The heat generated via GNP photothermal conversion was also reported to trigger drug (DOX) release from micellar GNPs and reverse drug resistance in the MCF-7 cell line [176]. Drug resistance reversal was also reported with another GNP polymeric hybrid; in this study, this resistance reversal was predicted to be due to increased heat-induced membrane fluidity [38]. Lin et al. also used GNP–micelle nanohybrids for pH-triggered drug release, but without involving any heat-induced drug liberation or PTT. In addition to pH-triggered drug release, this nanohybrid also improved CT imaging in vivo [177]. Likewise, folate-modified, DOX-loaded poly(L-aspartate)-b-poly(ethylene glycol) micellar GNPs carried and released DOX under acidic conditions, achieved higher cellular internalization via folate-mediated endocytosis and induced higher in vitro toxicity [178]. Furthermore, pH-responsive micelle–GNP hybrids were reported for dual PTT/chemotherapy delivery [179]. Micellar GNPs composed of a redox-responsive block copolymer and targeted via folic and lipoic acid were used for PTT, CT imaging, and the redox-triggered delivery of the drug GW627368X. This work reported an efficient active targeting of the micellar GNP hybrid, GSH-triggered drug release, and enhanced tumor cell death via PTT/chemotherapy synergy in vivo while retaining biosafety, as concluded following hemolysis studies [180]. Aryal et al. used micellar GNPs composed of GNPs and polycaprolactone-methoxy poly(ethylene glycol) for the untriggered delivery of 5-fluorouracil (chemotherapy) and reported controlled drug release in vitro. However, no viability studies were conducted [181]. Similar to [177], this study could be further improved by incorporating heat release triggers, PTT, or radiotherapy. It is important to note that GNP–micelle hybrids were also used to improve imaging, including photoacoustic [179,182] and CT imaging [183], thereby extending the potential of micellar GNPs even to image-guided therapy.

**Table 2:** Multimodal GNP–polymer nanohybrids for cancer therapy.

Hydrogel–GNP Hybrids						
Polymer	Triggering Stimuli	Targeted Cancer	Loaded Agents and Surface Modifications	Release Mechanisms	In Vitro/In Vivo Toxicity	Reference
Alginate	NIR-generated heat	Colon cancer	Cisplatin	Hyperthermia-triggered cisplatin release Chemotherapy, radiotherapy, and PTT synergy	Chemotherapy/radiotherapy/PTT synergy in vivo	[157]
Alginate	Fe_3_O_4_	Colon cancer	DOX Iron oxide nanoparticles	Magnetically guided chemotherapy/PTT Iron oxide-enhanced MRI	Chemotherapy/PTT synergy in vivo	[159]
Chitosan	NIR-generated heat	Melanoma	Liposomal DOX	Hyperthermia-triggered DOX release	Chemotherapy toxicity in vivo	[88]
Chitosan PNIPAM	Low pH NIR-generated heat	Breast cancer	Curcumin	Hyperthermia- and low-pH-triggered curcumin release Chemotherapy/PTT synergy	Chemotherapy/PTT synergy in vitro	[35]
Chitosan	Low pH NIR-generated heat	Breast cancer	DOX Porous silica nanoparticles	Hyperthermia- and low-pH-triggered curcumin release Chemotherapy/PTT synergy	Chemotherapy/PTT synergy in vivo	[160]
PNIPAAm Carboxymethyl chitosan	NIR-generated heat		DOX Iron oxide nanoparticles	Heat-triggered DOX release	Chemotherapy toxicity in vitro	[36]
PNIPAAm, HEMA, maleic acid, N,N’-bis(acryloyl)cystamine	Non-NIR-generated heat Redox Low pH		DOX 6-marcaptopurine PEG	pH-, redox-, and temperature-triggered drug release	Chemotherapy toxicity in vitro	[147]
DNA	NIR-generated heat	Melanoma	DOX	Hyperthermia-triggered DOX release Chemotherapy/PTT synergy	Chemotherapy/PTT synergy in vitro and in vivo	[165]
Hyaluronic acid	Hyaluronidase (enzyme) NIR-generated heat	Stomach cancer	DOX Triphenylphosphine	HA- and triphenylphosphine-mediated targeting Hyaluronidase and hyperthermia-triggered DOX release Chemotherapy/PTT synergy	Chemotherapy/PTT synergy in vitro and in vivo	[166]
Hyaluronic acid	NIR-generated heat GSH	Breast cancer	DOX	GSH- and hyperthermia-triggered DOX release HA-mediated targeting Drug resistance reversal in vitro, possibly due to enhanced hyperthermia-induced membrane fluidity	Chemotherapy/PTT synergy in vitro	[173]
**Micellar GNP hybrids**						
PLL	NIR-generated heat	Breast cancer	Paclitaxel	Hyperthermia-triggered paclitaxel release Chemotherapy/PTT synergy	Chemotherapy/PTT synergy in vitro and in vivo	[175]
PEG-b-PHEA	GSH	Cervical cancer	GW627368X Folic acid Lipoic acid	Folic and lipoic acid-mediated targeting Redox (GSH)-triggered GW627368X release Chemotherapy/PTT synergy	Chemotherapy/PTT synergy in vitro and in vivo	[180]
b-cyclodextrin-{poly(lactide)-poly(2-(d imethylamino) ethyl methacrylate)-poly[oligo(2-ethyl-2-oxazoline)methacrylate]}_21_ [b-CD-(PLAPDMAEMA-PEtOxMA)_21_]	Low pH	Liver cancer	DOX	Low-pH-triggered DOX release	Chemotherapy toxicity in vitro	[177]
poly(ethylene glycol)-b-poly(ε-caprolactone) (PEG- PCL-LA)	NIR-generated heat	Breast cancer	DOX	Hyperthermia-triggered DOX release Resistance reversal	Chemotherapy toxicity in vitro	[176]
poly(L-aspartate)-b-poly(ethylene glycol) copolymer	Low pH	Breast cancer	DOX Folic acid	FA-mediated targeting Low-pH-triggered DOX release	Chemotherapy toxicity in vitro	[178]
PEG-PAsp(DIP)-b-PAsp(MEA)	Low pH GSH NIR	Ovarian cancer	DOX	NIR-, low-pH-, and GSH-triggered DOX release PTT/chemotherapy synergy	Chemotherapy/PTT synergy in vitro and in vivo	[179]
poly(ethylene glycol)-block-poly(propylene glycol)-block-poly(ethylene glycol)	Low pH	Breast cancer	ZD6474 (dual tyrosine kinase inhibitor)	Low-pH-triggered ZD6474 release	Chemotherapy toxicity in vitro	[184]
**Other polymeric GNP hybrids**						
DNA	Low pH DNase II (nuclease)	Breast cancer	HER2 affibody 5-fluorouracil DOX	Low-pH- and DNase II-triggered drug release HER2-affibody-mediated targeted and internalization	DOX/5-fluorouracil synergy in vitro	[136]
PLGA	NIR-generated heat	Cervical cancer	DOX	Hyperthermia-improved DOX release Chemotherapy/PTT synergy	Chemotherapy/PTT synergy in vitro	[138]
Polyrotaxanes	NIR-generated heat	_______	DOX Cisplatin	Hyperthermia-triggered drug release	Chemotherapy toxicity in vitro	[33]

## 4. Challenges and Future Directions

**Figure 4:**
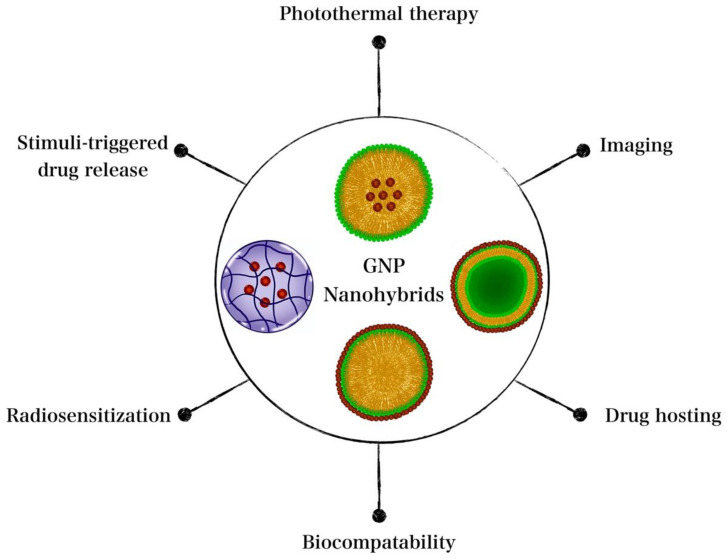
Advantages provided by GNP nanohybrids for cancer theragnostics.

The therapeutic performance of the GNP nanohybrids described in this review indicates their potential as multimodal therapeutic agents capable of chemotherapy, PTT, radiotherapy, and imaging. Furthermore, nanohybrids were not only thermoresponsive, but also multi-responsive in some cases. Some nanohybrids showed excellent therapeutic performance despite using unimodal approaches (e.g., single PTT). The potential of GNP nanohybrids as imaging agents was also evident [106,122,179,183,185], thereby providing an advantage for their future utilization for image-guided therapy. It is particularly interesting for a single nanocomposite to simultaneously hold several therapeutic features. The favorable properties provided by GNP nanohybrids for cancer theragnostic applications are summarized in Figure 4.

Generally, nanomaterials have advantages that make them suitable for clinical applications, such as their small size, which allows their blood circulation without blood flow disruption to be achieved. However, their bench-to-bedside translation is hindered by several practical obstacles [186,187,188]. Despite GNP nanohybrids’ potential, as with any other therapeutic formulation, discrepancies between the promising preclinical results and clinical outcomes are highly possible. It is important to point out that the success rates of the clinical transition of therapeutics, especially for cancer treatment, are notably low [189]. One potential way to reduce the chance of such discrepancies involves the use of patient-derived xenografts (PDXs). Immunodeficient mice injected with cancer cell lines fail to represent the molecular structure and heterogeneity associated with the original tumor. This leads to the preclinical–clinical inconsistency in the results seen with anti-tumor agents. PDXs involve directly implanting patient tumor fragments into immunocompromised mice, so that the tissues retain the original tumor cellular/histologic features, important stromal components, and gene expression profile. PDXs were found to closely match patient responses to treatments such as chemotherapy [190]. Therefore, utilizing PDXs could more accurately predict the clinical behavior of GNP nanohybrids and, thus, more accurately predict their efficiency as anti-tumor agents. This would make possible a fairer judgment of how worthy GNP nanohybrids are for cancer treatment at the patient level. Koga et al. well reviewed the use of PDXs as models of anti-cancer therapeutic formulations at the clinical stage [191]. 

Furthermore, some studies with GNP nanohybrids focused mainly on cancer cells while disregarding other important components of the TME. Cancer cells do not exist in isolation; rather, they exist within other components that they interact with to maintain their survival and growth. For example, cancer-associated fibroblasts are among the stromal components that play an important role in tumor progression and invasion. Therefore, it is important to explore and understand the interaction of the nanomaterial being studied with key non-cancerous, tumor-promoting TME components, such as cancer-associated fibroblasts [192]. For example, Bromma et al. [192] investigated the interaction of GNPs with two essential stromal components involved in cancer, fibroblasts, and cancer-associated fibroblasts. The investigation aimed to understand the fate of the nanoparticle within non-cancerous key TME components. It provided insights into inhibiting cancer growth by tackling both cancerous and non-cancerous constituents. 

Likewise, most of the hybrid GNPs studied for cancer theragnostics used EPR as a targeting mechanism. While some used ligand-mediated active targeting, the EPR effect is the basis of nanoformulations for cancer therapy [193]. Using EPR as a tumor-targeting mechanism raises another concern for future clinical applications of GNP nanohybrids due to EPR heterogeneity. EPR heterogeneity refers to the varying EPR effects exhibited by different tumors. For instance, while hepatocellular and renal carcinomas have a higher vascular density and thus higher EPR effect and higher drug accumulation, prostate and pancreatic cancers exhibit different characteristics [194]. The EPR effect varies between cancer types as well as between different stages of cancer, among patients having the same cancer, and even within the tumor itself [194,195]. Therefore, a good comprehension of this effect is necessary to optimize hybrid GNPs for treatment based on the specific cancer type and the patient. Further discussion on the effect of EPR heterogeneity on cancer treatment was provided by Maeda et al. [194]. 

Another concern with GNP hybrids is the general lack of analyses of interactions between the formulations and the blood components. Some interactions with blood components could have adverse effects on the normal functions of blood cells [196,197,198]. Therefore, for the further progression of GNP hybrids in cancer treatment, a more extensive analysis of blood–GNP hybrid interactions is needed. The International Organization of Standards (ISO) guideline for hemocompatibility testing recommends testing for thrombosis, coagulation, platelets, hematology, and immunology (complement and leukocytes). Furthermore, hemocompatibility testing depends not just on the material–blood interaction but also on other parameters, such as blood coagulability. It is important to note that in vivo hemocompatibility is hindered by differences among species, which may restrict the reliability of those results in an actual clinical setting [199]. 

Therefore, at this point, assuming the success of GNP nanohybrids as anti-cancer agents would be an overstatement due to the present clinical challenges and complexities. However, it is safe to say that GNP nanohybrids are promising in terms of preliminary results.

## 5. Conclusions

In conclusion, the potential of GNP hybrid nanostructures as multimodal cancer therapy agents is clear. GNP nanohybrids were found to kill tumor cells not only via triggered drug release, PTT, and radiotherapy but also via a combination of those strategies. In addition to the studies that utilized NIR-generated hyperthermia as a stimulus for triggering cargo release, several others did not use any stimulus yet reported to improve anti-tumor performance. This implies that further improved performance could be achieved by incorporating a release-triggering stimulus into the system. Furthermore, based on our search, radiotherapy has not been explored with liposomal GNPs. Other GNP nanohybrids, namely, polymeric GNP nanohybrids, were reported to improve radiotherapy and even combine it with other therapeutic strategies, such as chemotherapy and PTT [25]. This indicates the possibility of future incorporation of radiotherapy with PTT and chemotherapy in liposomal nanohybrids for more efficient tumor eradication.

GNP hybrids are promising imaging agents, so they could possibly provide a single platform for image-guided chemotherapy/PTT/radiotherapy. Based on the available literature, liposomal GNPs seem to be capable of responding to a single stimulus (heat), unless combined with another material, such as chitosan, which can respond to another stimulus (e.g., pH). Polymeric GNPs, on the other hand, seem to respond simultaneously to multiple stimuli, thereby making them more advantageous when it comes to tumor-specific drug release. This is due to the wide range of polymers that can make up the nanocarrier, each of which possesses different properties and stimulus responsivity. 

Although this review shows the exceptional performance of GNP hybrids for multimodal cancer therapy, these data are not enough to ensure their clinical effectiveness and efficiency. Preclinical–clinical result inconsistency is common among therapeutic formulations. One suggestion to better predict GNP hybrid nanostructures’ clinical efficiency involves performing studies with patient-derived xenografts for a better cellular, histologic, and genetic representation of tumors. Furthermore, extending biocompatibility testing to examine blood–GNP hybrid interactions is important. Although a few studies did conduct hemolysis assays, many others did not. Therefore, there is a need for the hemocompatibility testing of those GNP hybrid nanostructures. At the preliminary stage, GNP hybrids do hold a lot of potential as multimodal cancer therapeutics.

## Data Availability

Not applicable.

## References

[1] Sibuyi N.R.S., Moabelo K.L., Fadaka A.O., Meyer S., Onani M.O., Madiehe A.M., Meyer M. (2021). Multifunctional Gold Nanoparticles for Improved Diagnostic and Therapeutic Applications: A Review. Nanoscale Res. Lett..

[2] Kong F.-Y., Zhang J.-W., Li R.-F., Wang Z.-X., Wang W.-J., Wang W. (2017). Unique Roles of Gold Nanoparticles in Drug Delivery, Targeting and Imaging Applications. Molecules.

[3] Tiwari P., Vig K., Dennis V., Singh S. (2011). Functionalized Gold Nanoparticles and Their Biomedical Applications. Nanomaterials.

[4] Chen X., Li Q.W., Wang X.M. (2014). Gold Nanostructures for Bioimaging, Drug Delivery and Therapeutics. Precious Metals for Biomedical Applications.

[5] Chandran P.R., Thomas R.T. (2015). Gold Nanoparticles in Cancer Drug Delivery. Nanotechnology Applications for Tissue Engineering.

[6] Maccora D., Dini V., Battocchio C., Fratoddi I., Cartoni A., Rotili D., Castagnola M., Faccini R., Bruno I., Scotognella T. (2019). Gold Nanoparticles and Nanorods in Nuclear Medicine: A Mini Review. Appl. Sci..

[7] Rai M., Yadav A. (2019). Nanobiotechnology in Neurodegenerative Diseases.

[8] Rodzik-Czałka Ł., Lewandowska-Łańcucka J., Gatta V., Venditti I., Fratoddi I., Szuwarzyński M., Romek M., Nowakowska M. (2018). Nucleobases Functionalized Quantum Dots and Gold Nanoparticles Bioconjugates as a Fluorescence Resonance Energy Transfer (FRET) System—Synthesis, Characterization and Potential Applications. J. Colloid Interface Sci..

[9] Vines J.B., Yoon J.-H., Ryu N.-E., Lim D.-J., Park H. (2019). Gold Nanoparticles for Photothermal Cancer Therapy. Front. Chem..

[10] Sztandera K., Gorzkiewicz M., Klajnert-Maculewicz B. (2019). Gold Nanoparticles in Cancer Treatment. Mol. Pharm..

[11] Huang X., El-Sayed M.A. (2010). Gold Nanoparticles: Optical Properties and Implementations in Cancer Diagnosis and Photothermal Therapy. J. Adv. Res..

[12] Hu X., Zhang Y., Ding T., Liu J., Zhao H. (2020). Multifunctional Gold Nanoparticles: A Novel Nanomaterial for Various Medical Applications and Biological Activities. Front. Bioeng. Biotechnol..

[13] Yu C., Xu L., Zhang Y., Timashev P.S., Huang Y., Liang X.-J. (2020). Polymer-Based Nanomaterials for Noninvasive Cancer Photothermal Therapy. ACS Appl. Polym. Mater..

[14] Behrouzkia Z., Joveini Z., Keshavarzi B., Eyvazzadeh N., Aghdam R.Z. (2016). Hyperthermia: How Can It Be Used?. Oman Med. J..

[15] Dunne M., Regenold M., Allen C. (2020). Hyperthermia Can Alter Tumor Physiology and Improve Chemo- and Radio-Therapy Efficacy. Adv. Drug Deliv. Rev..

[16] Beik J., Abed Z., Ghoreishi F.S., Hosseini-Nami S., Mehrzadi S., Shakeri-Zadeh A., Kamrava S.K. (2016). Nanotechnology in Hyperthermia Cancer Therapy: From Fundamental Principles to Advanced Applications. J. Control. Release.

[17] Liu X., Zhang Y., Wang Y., Zhu W., Li G., Ma X., Zhang Y., Chen S., Tiwari S., Shi K. (2020). Comprehensive Understanding of Magnetic Hyperthermia for Improving Antitumor Therapeutic Efficacy. Theranostics.

[18] Petryk A.A., Giustini A.J., Gottesman R.E., Kaufman P.A., Hoopes P.J. (2013). Magnetic Nanoparticle Hyperthermia Enhancement of Cisplatin Chemotherapy Cancer Treatment. Int. J. Hyperth..

[19] Najahi-Missaoui W., Arnold R.D., Cummings B.S. (2020). Safe Nanoparticles: Are We There Yet?. IJMS.

[20] Jia Y.-P., Ma B.-Y., Wei X.-W., Qian Z.-Y. (2017). The in Vitro and in Vivo Toxicity of Gold Nanoparticles. Chin. Chem. Lett..

[21] Singh A.K., Srivastava O.N. (2015). One-Step Green Synthesis of Gold Nanoparticles Using Black Cardamom and Effect of PH on Its Synthesis. Nanoscale Res. Lett..

[22] Yang W., Xia B., Wang L., Ma S., Liang H., Wang D., Huang J. (2021). Shape Effects of Gold Nanoparticles in Photothermal Cancer Therapy. Mater. Today Sustain..

[23] Chithrani B.D., Ghazani A.A., Chan W.C.W. (2006). Determining the Size and Shape Dependence of Gold Nanoparticle Uptake into Mammalian Cells. Nano Lett..

[24] Alamzadeh Z., Beik J., Mirrahimi M., Shakeri-Zadeh A., Ebrahimi F., Komeili A., Ghalandari B., Ghaznavi H., Kamrava S.K., Moustakis C. (2020). Gold Nanoparticles Promote a Multimodal Synergistic Cancer Therapy Strategy by Co-Delivery of Thermo-Chemo-Radio Therapy. Eur. J. Pharm. Sci..

[25] Keshavarz M., Moloudi K., Paydar R., Abed Z., Beik J., Ghaznavi H., Shakeri-Zadeh A. (2018). Alginate Hydrogel Co-Loaded with Cisplatin and Gold Nanoparticles for Computed Tomography Image-Guided Chemotherapy. J. Biomater. Appl..

[26] Hainfeld J.F., Dilmanian F.A., Zhong Z., Slatkin D.N., Kalef-Ezra J.A., Smilowitz H.M. (2010). Gold Nanoparticles Enhance the Radiation Therapy of a Murine Squamous Cell Carcinoma. Phys. Med. Biol..

[27] Piccolo O., Lincoln J.D., Melong N., Orr B.C., Fernandez N.R., Borsavage J., Berman J.N., Robar J., Ha M.N. (2022). Radiation Dose Enhancement Using Gold Nanoparticles with a Diamond Linear Accelerator Target: A Multiple Cell Type Analysis. Sci. Rep..

[28] Popovtzer R., Agrawal A., Kotov N.A., Popovtzer A., Balter J., Carey T.E., Kopelman R. (2008). Targeted Gold Nanoparticles Enable Molecular CT Imaging of Cancer. Nano Lett..

[29] Chemla Y., Betzer O., Markus A., Farah N., Motiei M., Popovtzer R., Mandel Y. (2019). Gold Nanoparticles for Multimodal High-Resolution Imaging of Transplanted Cells for Retinal Replacement Therapy. Nanomedicine.

[30] Mirrahimi M., Khateri M., Beik J., Ghoreishi F.S., Dezfuli A.S., Ghaznavi H., Shakeri-Zadeh A. (2019). Enhancement of Chemoradiation by Co-incorporation of Gold Nanoparticles and Cisplatin into Alginate Hydrogel. J. Biomed. Mater. Res..

[31] Hsiao C.-W., Chuang E.-Y., Chen H.-L., Wan D., Korupalli C., Liao Z.-X., Chiu Y.-L., Chia W.-T., Lin K.-J., Sung H.-W. (2015). Photothermal Tumor Ablation in Mice with Repeated Therapy Sessions Using NIR-Absorbing Micellar Hydrogels Formed in Situ. Biomaterials.

[32] Tan C., Chen J., Wu X.-J., Zhang H. (2018). Epitaxial Growth of Hybrid Nanostructures. Nat. Rev. Mater..

[33] Adeli M., Sarabi R.S., Yadollahi Farsi R., Mahmoudi M., Kalantari M. (2011). Polyrotaxane/Gold Nanoparticle Hybrid Nanomaterials as Anticancer Drug Delivery Systems. J. Mater. Chem..

[34] Koga K., Tagami T., Ozeki T. (2021). Gold Nanoparticle-Coated Thermosensitive Liposomes for the Triggered Release of Doxorubicin, and Photothermal Therapy Using a near-Infrared Laser. Colloids Surf. A Physicochem. Eng. Asp..

[35] Howaili F., Özliseli E., Küçüktürkmen B., Razavi S.M., Sadeghizadeh M., Rosenholm J.M. (2021). Stimuli-Responsive, Plasmonic Nanogel for Dual Delivery of Curcumin and Photothermal Therapy for Cancer Treatment. Front. Chem..

[36] Pourjavadi A., Doroudian M., Bagherifard M., Bahmanpour M. (2020). Magnetic and Light-Responsive Nanogels Based on Chitosan Functionalized with Au Nanoparticles and Poly(N-Isopropylacrylamide) as a Remotely Triggered Drug Carrier. New J. Chem..

[37] Yang L. (2017). Tumor Microenvironment and Metabolism. IJMS.

[38] Xing S., Zhang X., Luo L., Cao W., Li L., He Y., An J., Gao D. (2018). Doxorubicin/Gold Nanoparticles Coated with Liposomes for Chemo-Photothermal Synergetic Antitumor Therapy. Nanotechnology.

[39] Wang M., Liu Y., Zhang X., Luo L., Li L., Xing S., He Y., Cao W., Zhu R., Gao D. (2017). Gold Nanoshell Coated Thermo-PH Dual Responsive Liposomes for Resveratrol Delivery and Chemo-Photothermal Synergistic Cancer Therapy. J. Mater. Chem. B.

[40] Alle M., Sharma G., Lee S.-H., Kim J.-C. (2022). Next-Generation Engineered Nanogold for Multimodal Cancer Therapy and Imaging: A Clinical Perspectives. J. Nanobiotechnol..

[41] Mauro N., Utzeri M.A., Varvarà P., Cavallaro G. (2021). Functionalization of Metal and Carbon Nanoparticles with Potential in Cancer Theranostics. Molecules.

[42] Debela D.T., Muzazu S.G., Heraro K.D., Ndalama M.T., Mesele B.W., Haile D.C., Kitui S.K., Manyazewal T. (2021). New Approaches and Procedures for Cancer Treatment: Current Perspectives. SAGE Open Med..

[43] Bao Q.-Y., Zhang N., Geng D.-D., Xue J.-W., Merritt M., Zhang C., Ding Y. (2014). The Enhanced Longevity and Liver Targetability of Paclitaxel by Hybrid Liposomes Encapsulating Paclitaxel-Conjugated Gold Nanoparticles. Int. J. Pharm..

[44] Wang W., Shao A., Zhang N., Fang J., Ruan J.J., Ruan B.H. (2017). Cationic Polymethacrylate-Modified Liposomes Significantly Enhanced Doxorubicin Delivery and Antitumor Activity. Sci. Rep..

[45] Chowdhury N., Chaudhry S., Hall N., Olverson G., Zhang Q.-J., Mandal T., Dash S., Kundu A. (2020). Targeted Delivery of Doxorubicin Liposomes for Her-2+ Breast Cancer Treatment. AAPS Pharm. Sci. Tech..

[46] Zhang X., Zhu T., Miao Y., Zhou L., Zhang W. (2020). Dual-Responsive Doxorubicin-Loaded Nanomicelles for Enhanced Cancer Therapy. J. Nanobiotechnol..

[47] Norouzi M., Yathindranath V., Thliveris J.A., Kopec B.M., Siahaan T.J., Miller D.W. (2020). Doxorubicin-Loaded Iron Oxide Nanoparticles for Glioblastoma Therapy: A Combinational Approach for Enhanced Delivery of Nanoparticles. Sci. Rep..

[48] An X. (2017). Stimuli-Responsive Liposome and Control Release Drug.

[49] Wang D., Green M.D., Chen K., Daengngam C., Kotsuchibashi Y. (2016). Stimuli-Responsive Polymers: Design, Synthesis, Characterization, and Applications. Int. J. Polym. Sci..

[50] Ortega-García A., Martínez-Bernal B.G., Ceja I., Mendizábal E., Puig-Arévalo J.E., Pérez-Carrillo L.A. (2022). Drug Delivery from Stimuli-Responsive Poly(N-Isopropylacrylamide-Co-N-Isopropylmethacrylamide)/Chitosan Core/Shell Nanohydrogels. Polymers.

[51] Harris M., Ahmed H., Barr B., LeVine D., Pace L., Mohapatra A., Morshed B., Bumgardner J.D., Jennings J.A. (2017). Magnetic Stimuli-Responsive Chitosan-Based Drug Delivery Biocomposite for Multiple Triggered Release. Int. J. Biol. Macromol..

[52] Hajebi S., Rabiee N., Bagherzadeh M., Ahmadi S., Rabiee M., Roghani-Mamaqani H., Tahriri M., Tayebi L., Hamblin M.R. (2019). Stimulus-Responsive Polymeric Nanogels as Smart Drug Delivery Systems. Acta Biomater..

[53] Hossen S., Hossain M.K., Basher M.K., Mia M.N.H., Rahman M.T., Uddin M.J. (2019). Smart Nanocarrier-Based Drug Delivery Systems for Cancer Therapy and Toxicity Studies: A Review. J. Adv. Res..

[54] Venditti I., Testa G., Sciubba F., Carlini L., Porcaro F., Meneghini C., Mobilio S., Battocchio C., Fratoddi I. (2017). Hydrophilic Metal Nanoparticles Functionalized by 2-Diethylaminoethanethiol: A Close Look at the Metal−Ligand Interaction and Interface Chemical Structure. J. Phys. Chem. C.

[55] Yin X., Russek S.E., Zabow G., Sun F., Mohapatra J., Keenan K.E., Boss M.A., Zeng H., Liu J.P., Viert A. (2018). Large T1 Contrast Enhancement Using Superparamagnetic Nanoparticles in Ultra-Low Field MRI. Sci. Rep..

[56] Luo D., Wang X., Burda C., Basilion J.P. (2021). Recent Development of Gold Nanoparticles as Contrast Agents for Cancer Diagnosis. Cancers.

[57] Fan M., Han Y., Gao S., Yan H., Cao L., Li Z., Liang X.-J., Zhang J. (2020). Ultrasmall Gold Nanoparticles in Cancer Diagnosis and Therapy. Theranostics.

[58] Fontes de Paula Aguiar M., Bustamante Mamani J., Klei Felix T., Ferreira dos Reis R., Rodrigues da Silva H., Nucci L.P., Nucci-da-Silva M.P., Gamarra L.F. (2017). Magnetic Targeting with Superparamagnetic Iron Oxide Nanoparticles for In Vivo Glioma. Nanotechnol. Rev..

[59] Venditti I., Iucci G., Fratoddi I., Cipolletti M., Montalesi E., Marino M., Secchi V., Battocchio C. (2020). Direct Conjugation of Resveratrol on Hydrophilic Gold Nanoparticles: Structural and Cytotoxic Studies for Biomedical Applications. Nanomaterials.

[60] AlSawaftah N.M., Awad N.S., Pitt W.G., Husseini G.A. (2022). PH-Responsive Nanocarriers in Cancer Therapy. Polymers.

[61] Chauhan A. (2018). Dendrimers for Drug Delivery. Molecules.

[62] Choudhary S., Gupta L., Rani S., Dave K., Gupta U. (2017). Impact of Dendrimers on Solubility of Hydrophobic Drug Molecules. Front. Pharmacol..

[63] de Lima C.S.A., Balogh T.S., Varca J.P.R.O., Varca G.H.C., Lugão A.B., Camacho-Cruz L.A., Bucio E., Kadlubowski S.S. (2020). An Updated Review of Macro, Micro, and Nanostructured Hydrogels for Biomedical and Pharmaceutical Applications. Pharmaceutics.

[64] Chai Q., Jiao Y., Yu X. (2017). Hydrogels for Biomedical Applications: Their Characteristics and the Mechanisms behind Them. Gels.

[65] Kopeček J., Yang J. (2007). Hydrogels as Smart Biomaterials. Polym. Int..

[66] Santander-Ortega M.J., Lozano M.V., Uchegbu I.F., Schätzlein A.G. (2016). Dendrimers for Gene Therapy. Polymers and Nanomaterials for Gene Therapy.

[67] Lv J., Wang C., Li H., Li Z., Fan Q., Zhang Y., Li Y., Wang H., Cheng Y. (2020). Bifunctional and Bioreducible Dendrimer Bearing a Fluoroalkyl Tail for Efficient Protein Delivery Both In Vitro and In Vivo. Nano Lett..

[68] Liu C., Wan T., Wang H., Zhang S., Ping Y., Cheng Y. (2019). A Boronic Acid–Rich Dendrimer with Robust and Unprecedented Efficiency for Cytosolic Protein Delivery and CRISPR-Cas9 Gene Editing. Sci. Adv..

[69] Kandil R., Merkel O.M. (2019). Recent Progress of Polymeric Nanogels for Gene Delivery. Curr. Opin. Colloid Interface Sci..

[70] Kousalová J., Etrych T. (2018). Polymeric Nanogels as Drug Delivery Systems. Physiol. Res..

[71] Xu X., Shen S., Mo R. (2022). Bioresponsive Nanogels for Protein Delivery. View.

[72] Wang H., Huang Q., Chang H., Xiao J., Cheng Y. (2016). Stimuli-Responsive Dendrimers in Drug Delivery. Biomater. Sci..

[73] Chacko R.T., Ventura J., Zhuang J., Thayumanavan S. (2012). Polymer Nanogels: A Versatile Nanoscopic Drug Delivery Platform. Adv. Drug Deliv. Rev..

[74] Bahutair W.N., Abuwatfa W.H., Husseini G.A. (2022). Ultrasound Triggering of Liposomal Nanodrugs for Cancer Therapy: A Review. Nanomaterials.

[75] Hanafy N., El-Kemary M., Leporatti S. (2018). Micelles Structure Development as a Strategy to Improve Smart Cancer Therapy. Cancers.

[76] Ghezzi M., Pescina S., Padula C., Santi P., Del Favero E., Cantù L., Nicoli S. (2021). Polymeric Micelles in Drug Delivery: An Insight of the Techniques for Their Characterization and Assessment in Biorelevant Conditions. J. Control. Release.

[77] Jhaveri A.M., Torchilin V.P. (2014). Multifunctional Polymeric Micelles for Delivery of Drugs and SiRNA. Front. Pharmacol..

[78] Zhou Q., Zhang L., Yang T., Wu H. (2018). Stimuli-Responsive Polymeric Micelles for Drug Delivery and Cancer Therapy. IJN.

[79] Mi P. (2020). Stimuli-Responsive Nanocarriers for Drug Delivery, Tumor Imaging, Therapy and Theranostics. Theranostics.

[80] Ou Y.-C., Webb J.A., Faley S., Shae D., Talbert E.M., Lin S., Cutright C.C., Wilson J.T., Bellan L.M., Bardhan R. (2016). Gold Nanoantenna-Mediated Photothermal Drug Delivery from Thermosensitive Liposomes in Breast Cancer. ACS Omega.

[81] Luo L., Bian Y., Liu Y., Zhang X., Wang M., Xing S., Li L., Gao D. (2016). Combined Near Infrared Photothermal Therapy and Chemotherapy Using Gold Nanoshells Coated Liposomes to Enhance Antitumor Effect. Small.

[82] He H., Liu L., Zhang S., Zheng M., Ma A., Chen Z., Pan H., Zhou H., Liang R., Cai L. (2020). Smart Gold Nanocages for Mild Heat-Triggered Drug Release and Breaking Chemoresistance. J. Control. Release.

[83] Li Y., He D., Tu J., Wang R., Zu C., Chen Y., Yang W., Shi D., Webster T.J., Shen Y. (2018). Comparative Effect of Wrapping Solid Gold Nanoparticles and Hollow Gold Nanoparticles with Doxorubicin-Loaded Thermosensitive Liposomes for Cancer Thermo-Chemotherapy. Nanoscale.

[84] Hossann M., Kneidl B., Peller M., Lindner L., Winter G. (2014). Thermosensitive Liposomal Drug Delivery Systems: State of the Art Review. IJN.

[85] Sercombe L., Veerati T., Moheimani F., Wu S.Y., Sood A.K., Hua S. (2015). Advances and Challenges of Liposome Assisted Drug Delivery. Front. Pharmacol..

[86] Olusanya T., Haj Ahmad R., Ibegbu D., Smith J., Elkordy A. (2018). Liposomal Drug Delivery Systems and Anticancer Drugs. Molecules.

[87] Bhardwaj V., Kaushik A., Khatib Z.M., Nair M., McGoron A.J. (2019). Recalcitrant Issues and New Frontiers in Nano-Pharmacology. Front. Pharmacol..

[88] Won J.E., Wi T.I., Lee C.M., Lee J.H., Kang T.H., Lee J.-W., Shin B.C., Lee Y., Park Y.-M., Han H.D. (2021). NIR Irradiation-Controlled Drug Release Utilizing Injectable Hydrogels Containing Gold-Labeled Liposomes for the Treatment of Melanoma Cancer. Acta Biomater..

[89] Lorusso D., Di Stefano A., Carone V., Fagotti A., Pisconti S., Scambia G. (2007). Pegylated Liposomal Doxorubicin-Related Palmar-Plantar Erythrodysesthesia (‘Hand-Foot’ Syndrome). Ann. Oncol..

[90] Seynhaeve A.L.B. (2013). Intact Doxil Is Taken up Intracellularly and Released Doxorubicin Sequesters in the Lysosome: Evaluated by in Vitro/in Vivo Live Cell Imaging. J. Control. Release.

[91] Chaikomon K., Chattong S., Chaiya T., Tiwawech D., Sritana-anant Y., Sereemaspun A., Manotham K. (2018). Doxorubicin-Conjugated Dexamethasone Induced MCF-7 Apoptosis without Entering the Nucleus and Able to Overcome MDR-1-Induced Resistance. DDDT.

[92] Thakur N.S., Patel G., Kushwah V., Jain S., Banerjee U.C. (2019). Self-Assembled Gold Nanoparticle−Lipid Nanocomposites for On- Demand Delivery, Tumor Accumulation, and Combined Photothermal−Photodynamic Therapy. ACS Appl. Bio Mater..

[93] Kang Z., Yan X., Zhao L., Liao Q., Zhao K., Du H., Zhang X., Zhang X., Zhang Y. (2015). Gold Nanoparticle/ZnO Nanorod Hybrids for Enhanced Reactive Oxygen Species Generation and Photodynamic Therapy. Nano Res..

[94] Zhao T., Yu K., Li L., Zhang T., Guan Z., Gao N., Yuan P., Li S., Yao S.Q., Xu Q.-H. (2014). Gold Nanorod Enhanced Two-Photon Excitation Fluorescence of Photosensitizers for Two-Photon Imaging and Photodynamic Therapy. ACS Appl. Mater. Interfaces.

[95] Srivatsan A., Jenkins S.V., Jeon M., Wu Z., Kim C., Chen J., Pandey R. (2014). Gold Nanocage-Photosensitizer Conjugates for Dual-Modal Image-Guided Enhanced Photodynamic Therapy. Theranostics.

[96] Kautzka Z., Clement S., Goldys E.M., Deng W. (2017). Light-Triggered Liposomal Cargo Delivery Platform Incorporating Photosensitizers and Gold Nanoparticles for Enhanced Singlet Oxygen Generation and Increased Cytotoxicity. IJN.

[97] Singh S.P., Alvi S.B., Pemmaraju D.B., Singh A.D., Manda S.V., Srivastava R., Rengan A.K. (2018). NIR Triggered Liposome Gold Nanoparticles Entrapping Curcumin as in Situ Adjuvant for Photothermal Treatment of Skin Cancer. Int. J. Biol. Macromol..

[98] Liu Y., Zhang X., Luo L., Li L., Zhu R.Y., Li A., He Y., Cao W., Niu K., Liu H. (2019). Gold-Nanobranched-Shell Based Drug Vehicles with Ultrahigh Photothermal Efficiency for Chemo-Photothermal Therapy. Nanomed. Nanotechnol. Biol. Med..

[99] Chauhan D.S., Prasad R., Devrukhkar J., Selvaraj K., Srivastava R. (2018). Disintegrable NIR Light Triggered Gold Nanorods Supported Liposomal Nanohybrids for Cancer Theranostics. Bioconjugate Chem..

[100] Liu Y., Zhang X., Liu Z., Wang L., Luo L., Wang M., Wang Q., Gao D. (2017). Gold Nanoshell-Based Betulinic Acid Liposomes for Synergistic Chemo-Photothermal Therapy. Nanomed. Nanotechnol. Biol. Med..

[101] Hua H., Zhang N., Liu D., Song L., Liu T., Li S., Zhao Y. (2017). Multifunctional Gold Nanorods and Docetaxel-Encapsulated Liposomes for Combined Thermo- and Chemotherapy. IJN.

[102] Nguyen V.D., Min H.-K., Kim C.-S., Han J., Park J.-O., Choi E. (2019). Folate Receptor-Targeted Liposomal Nanocomplex for Effective Synergistic Photothermal-Chemotherapy of Breast Cancer in Vivo. Colloids Surf. B Biointerfaces.

[103] You J., Zhang P., Hu F., Du Y., Yuan H., Zhu J., Wang Z., Zhou J., Li C. (2014). Near-Infrared Light-Sensitive Liposomes for the Enhanced Photothermal Tumor Treatment by the Combination with Chemotherapy. Pharm. Res..

[104] Li Y., Song W., Hu Y., Xia Y., Li Z., Lu Y., Shen Y. (2021). “Petal-like” Size-Tunable Gold Wrapped Immunoliposome to Enhance Tumor Deep Penetration for Multimodal Guided Two-Step Strategy. J. Nanobiotechnol..

[105] Zhang N., Li J., Hou R., Zhang J., Wang P., Liu X., Zhang Z. (2017). Bubble-Generating Nano-Lipid Carriers for Ultrasound/CT Imaging-Guided Efficient Tumor Therapy. Int. J. Pharm..

[106] Rengan A.K., Jagtap M., De A., Banerjee R., Srivastava R. (2014). Multifunctional Gold Coated Thermo-Sensitive Liposomes for Multimodal Imaging and Photo-Thermal Therapy of Breast Cancer Cells. Nanoscale.

[107] Park J.-M., Choi H.E., Kudaibergen D., Kim J.-H., Kim K.S. (2021). Recent Advances in Hollow Gold Nanostructures for Biomedical Applications. Front. Chem..

[108] You J., Zhang G., Li C. (2010). Exceptionally High Payload of Doxorubicin in Hollow Gold Nanospheres for Near-Infrared Light-Triggered Drug Release. ACS Nano.

[109] Xiong C., Lu W., Zhou M., Wen X., Li C. (2018). Cisplatin-Loaded Hollow Gold Nanoparticles for Laser-Triggered Release. Cancer Nano.

[110] Sonkar R., Sonali, Jha A., Viswanadh M.K., Burande A.S., Narendra, Pawde D.M., Patel K.K., Singh M., Koch B. (2021). Gold Liposomes for Brain-Targeted Drug Delivery: Formulation and Brain Distribution Kinetics. Mater. Sci. Eng. C.

[111] Kirui D.K., Celia C., Molinaro R., Bansal S.S., Cosco D., Fresta M., Shen H., Ferrari M. (2015). Mild Hyperthermia Enhances Transport of Liposomal Gemcitabine and Improves In Vivo Therapeutic Response. Adv. Healthc. Mater..

[112] Zhang N., Chen H., Liu A.-Y., Shen J.-J., Shah V., Zhang C., Hong J., Ding Y. (2016). Gold Conjugate-Based Liposomes with Hybrid Cluster Bomb Structure for Liver Cancer Therapy. Biomaterials.

[113] Hamzawy M.A., Abo-youssef A.M., Salem H.F., Mohammed S.A. (2017). Antitumor Activity of Intratracheal Inhalation of Temozolomide (TMZ) Loaded into Gold Nanoparticles and/or Liposomes against Urethane-Induced Lung Cancer in BALB/c Mice. Drug Deliv..

[114] Deng W., Chen W., Clement S., Guller A., Zhao Z., Engel A., Goldys E.M. (2018). Controlled Gene and Drug Release from a Liposomal Delivery Platform Triggered by X-Ray Radiation. Nat. Commun..

[115] Noh J., Kwon B., Han E., Park M., Yang W., Cho W., Yoo W., Khang G., Lee D. (2015). Amplification of Oxidative Stress by a Dual Stimuli-Responsive Hybrid Drug Enhances Cancer Cell Death. Nat. Commun..

[116] Wei X., Liao J., Davoudi Z., Zheng H., Chen J., Li D., Xiong X., Yin Y., Yu X., Xiong J. (2018). Folate Receptor-Targeted and GSH-Responsive Carboxymethyl Chitosan Nanoparticles Containing Covalently Entrapped 6-Mercaptopurine for Enhanced Intracellular Drug Delivery in Leukemia. Mar. Drugs.

[117] Jeon M., Kim G., Lee W., Baek S., Jung H.N., Im H.-J. (2021). Development of Theranostic Dual-Layered Au-Liposome for Effective Tumor Targeting and Photothermal Therapy. J. Nanobiotechnol..

[118] Kwon H.J., Byeon Y., Jeon H.N., Cho S.H., Han H.D., Shin B.C. (2015). Gold Cluster-Labeled Thermosensitive Liposmes Enhance Triggered Drug Release in the Tumor Microenvironment by a Photothermal Effect. J. Control. Release.

[119] Du B., Gu X., Han X., Ding G., Wang Y., Li D., Wang E., Wang J. (2017). Lipid-Coated Gold Nanoparticles Functionalized by Folic Acid as Gene Vectors for Targeted Gene Delivery in Vitro and in Vivo. ChemMedChem.

[120] Refaat A., del Rosal B., Palasubramaniam J., Pietersz G., Wang X., Moulton S.E., Peter K. (2021). Near-Infrared Light-Responsive Liposomes for Protein Delivery: Towards Bleeding-Free Photothermally-Assisted Thrombolysis. J. Control. Release.

[121] Grafals-Ruiz N., Rios-Vicil C.I., Lozada-Delgado E.L., Quiñones-Díaz B.I., Noriega-Rivera R.A., Martínez-Zayas G., Santana-Rivera Y., Santiago-Sánchez G.S., Valiyeva F., Vivas-Mejía P.E. (2020). Brain Targeted Gold Liposomes Improve RNAi Delivery for Glioblastoma. IJN.

[122] Jia X., Lv M., Fei Y., Dong Q., Wang H., Liu Q., Li D., Wang J., Wang E. (2022). Facile One-Step Synthesis of NIR-Responsive SiRNA-Inorganic Hybrid Nanoplatform for Imaging-Guided Photothermal and Gene Synergistic Therapy. Biomaterials.

[123] Skalickova S., Nejdl L., Kudr J., Ruttkay-Nedecky B., Jimenez Jimenez A., Kopel P., Kremplova M., Masarik M., Stiborova M., Eckschlager T. (2016). Fluorescence Characterization of Gold Modified Liposomes with Antisense N-Myc DNA Bound to the Magnetisable Particles with Encapsulated Anticancer Drugs (Doxorubicin, Ellipticine and Etoposide). Sensors.

[124] An S.S., Kang J.H., Ko Y.T. (2015). Lipid-Coated Gold Nanocomposites for Enhanced Cancer Therapy. IJN.

[125] Kunjiappan S., Panneerselvam T., Somasundaram B., Arunachalam S., Sankaranarayanan M., Parasuraman P. (2018). Preparation of Liposomes Encapsulated Epirubicin-Gold Nanoparticles for Tumor Specific Delivery and Release. Biomed. Phys. Eng. Express.

[126] Guité-Verret A., Vachon M. (2021). The Incurable Metastatic Breast Cancer Experience through Metaphors: The Fight and the Unveiling. Int. J. Qual. Stud. Health Well-Being.

[127] Ferlay J., Colombet M., Soerjomataram I., Parkin D.M., Piñeros M., Znaor A., Bray F. (2021). Cancer Statistics for the Year 2020: An Overview. Int. J. Cancer.

[128] Westphal T., Gampenrieder S.P., Rinnerthaler G., Greil R. (2018). Cure in Metastatic Breast Cancer. Memo.

[129] Prasad R., Jain N.K., Yadav A.S., Chauhan D.S., Devrukhkar J., Kumawat M.K., Shinde S., Gorain M., Thakor A.S., Kundu G.C. (2020). Liposomal Nanotheranostics for Multimode Targeted in Vivo Bioimaging and Near-infrared Light Mediated Cancer Therapy. Commun. Biol..

[130] Qin G., Li Z., Xia R., Li F., O’Neill B.E., Goodwin J.T., Khant H.A., Chiu W., Li K.C. (2011). Partially Polymerized Liposomes: Stable against Leakage yet Capable of Instantaneous Release for Remote Controlled Drug Delivery. Nanotechnology.

[131] García M.C., Calderón-Montaño J.M., Rueda M., Longhi M., Rabasco A.M., López-Lázaro M., Prieto-Dapena F., González-Rodríguez M.L. (2022). PH-Temperature Dual-Sensitive Nucleolipid-Containing Stealth Liposomes Anchored with PEGylated AuNPs for Triggering Delivery of Doxorubicin. Int. J. Pharm..

[132] Vigata M., Meinert C., Hutmacher D.W., Bock N. (2020). Hydrogels as Drug Delivery Systems: A Review of Current Characterization and Evaluation Techniques. Pharmaceutics.

[133] Das S.S., Bharadwaj P., Bilal M., Barani M., Rahdar A., Taboada P., Bungau S., Kyzas G.Z. (2020). Stimuli-Responsive Polymeric Nanocarriers for Drug Delivery, Imaging, and Theragnosis. Polymers.

[134] Thelu H.V.P., Albert S.K., Golla M., Krishnan N., Ram D., Srinivasula S.M., Varghese R. (2018). Size Controllable DNA Nanogels from the Self-Assembly of DNA Nanostructures through Multivalent Host–Guest Interactions. Nanoscale.

[135] Yao C., Yuan Y., Yang D. (2018). Magnetic DNA Nanogels for Targeting Delivery and Multistimuli-Triggered Release of Anticancer Drugs. ACS Appl. Bio Mater..

[136] Zhang C. (2020). Co-Delivery of 5-Fluorodeoxyuridine and Doxorubicin via Gold Nanoparticle Equipped with Affibody-DNA Hybrid Strands for Targeted Synergistic Chemotherapy of HER2 Overexpressing Breast Cancer. Sci. Rep..

[137] Kumar S., Peng X., Daley J., Yang L., Shen J., Nguyen N., Bae G., Niu H., Peng Y., Hsieh H.-J. (2017). Inhibition of DNA2 Nuclease as a Therapeutic Strategy Targeting Replication Stress in Cancer Cells. Oncogenesis.

[138] Park H., Yang J., Lee J., Haam S., Choi I.-H., Yoo K.-H. (2009). Multifunctional Nanoparticles for Combined Doxorubicin and Photothermal Treatments. ACS Nano.

[139] Dai M., Ja F. (2016). Engineered Protein Polymer-Gold Nanoparticle Hybrid Materials for Small Molecule Delivery. J. Nanomed. Nanotechnol..

[140] Narayanaswamy R., Torchilin V.P. (2019). Hydrogels and Their Applications in Targeted Drug Delivery. Molecules.

[141] Hoare T.R., Kohane D.S. (2008). Hydrogels in Drug Delivery: Progress and Challenges. Polymer.

[142] Cuggino J.C., Blanco E.R.O., Gugliotta L.M., Alvarez Igarzabal C.I., Calderón M. (2019). Crossing Biological Barriers with Nanogels to Improve Drug Delivery Performance. J. Control. Release.

[143] Soleimani K., Arkan E., Derakhshankhah H., Haghshenas B., Jahanban-Esfahlan R., Jaymand M. (2021). A Novel Bioreducible and PH-Responsive Magnetic Nanohydrogel Based on β-Cyclodextrin for Chemo/Hyperthermia Therapy of Cancer. Carbohydr. Polym..

[144] Häring M., Schiller J., Mayr J., Grijalvo S., Eritja R., Díaz D. (2015). Magnetic Gel Composites for Hyperthermia Cancer Therapy. Gels.

[145] Chiang W.-H., Ho V.T., Chen H.-H., Huang W.-C., Huang Y.-F., Lin S.-C., Chern C.-S., Chiu H.-C. (2013). Superparamagnetic Hollow Hybrid Nanogels as a Potential Guidable Vehicle System of Stimuli-Mediated MR Imaging and Multiple Cancer Therapeutics. Langmuir.

[146] Jin R.-M., Yao M.-H., Yang J., Zhao D.-H., Zhao Y.-D., Liu B. (2017). One-Step in Situ Synthesis of Polypeptide–Gold Nanoparticles Hybrid Nanogels and Their Application in Targeted Photoacoustic Imaging. ACS Sustain. Chem. Eng..

[147] Ghorbani M., Hamishehkar H. (2018). A Novel Multi Stimuli-Responsive PEGylated Hybrid Gold/Nanogels for Co-Delivery of Doxorubicin and 6-mercaptopurine. Mater. Sci. Eng. C.

[148] Zhang H., Zhu Y., Qu L., Wu H., Kong H., Yang Z., Chen D., Mäkilä E., Salonen J., Santos H.A. (2018). Gold Nanorods Conjugated Porous Silicon Nanoparticles Encapsulated in Calcium Alginate Nano Hydrogels Using Microemulsion Templates. Nano Lett..

[149] Wang D., Wu H., Zhou J., Xu P., Wang C., Shi R., Wang H., Wang H., Guo Z., Chen Q. (2018). In Situ One-Pot Synthesis of MOF-Polydopamine Hybrid Nanogels with Enhanced Photothermal Effect for Targeted Cancer Therapy. Adv. Sci..

[150] Zhang Q., Wu J., Wang J., Wang X., Wu C., Chen M., Wu Q., Lesniak M.S., Mi Y., Cheng Y. (2020). A Neutrophil-Inspired Supramolecular Nanogel for Magnetocaloric–Enzymatic Tandem Therapy. Angew. Chem. Int. Ed..

[151] Qu Y., Chu B., Wei X., Lei M., Hu D., Zha R., Zhong L., Wang M., Wang F., Qian Z. (2019). Redox/PH Dual-Stimuli Responsive Camptothecin Prodrug Nanogels for “on-Demand” Drug Delivery. J. Control. Release.

[152] Gao W., Zhang Y., Zhang Q., Zhang L. (2016). Nanoparticle-Hydrogel: A Hybrid Biomaterial System for Localized Drug Delivery. Ann. Biomed. Eng..

[153] Kiseleva M., Omar M.M., Boisselier É., Selivanova S.V., Fortin M.-A. (2022). A Three-Dimensional Printable Hydrogel Formulation for the Local Delivery of Therapeutic Nanoparticles to Cervical Cancer. ACS Biomater. Sci. Eng..

[154] Jeong B., Kim S.W., Bae Y.H. (2012). Thermosensitive Sol–Gel Reversible Hydrogels. Adv. Drug Deliv. Rev..

[155] Liu M., Huang P., Wang W., Feng Z., Zhang J., Deng L., Dong A. (2016). Injectable Nanocomposite Hydrogel Co-Constructed by Gold Nanorods and Paclitaxel-Loaded Nanoparticles for Local Chemo- Photothermal Synergetic Cancer Therapy. J. Mater. Chem. B.

[156] Lee K.Y., Mooney D.J. (2012). Alginate: Properties and Biomedical Applications. Prog. Polym. Sci..

[157] Mirrahimi M., Beik J., Mirrahimi M., Alamzadeh Z., Teymouri S., Mahabadi V.P., Eslahi N., Ebrahimi Tazehmahalleh F., Ghaznavi H., Shakeri-Zadeh A. (2020). Triple Combination of Heat, Drug and Radiation Using Alginate Hydrogel Co-Loaded with Gold Nanoparticles and Cisplatin for Locally Synergistic Cancer Therapy. Int. J. Biol. Macromol..

[158] Alamzadeh Z., Beik J., Pirhajati Mahabadi V., Abbasian Ardekani A., Ghader A., Kamrava S.K., Shiralizadeh Dezfuli A., Ghaznavi H., Shakeri-Zadeh A. (2019). Ultrastructural and Optical Characteristics of Cancer Cells Treated by a Nanotechnology Based Chemo-Photothermal Therapy Method. J. Photochem. Photobiol. B Biol..

[159] Khani T., Alamzadeh Z., Sarikhani A., Mousavi M., Mirrahimi M., Tabei M., Irajirad R., Abed Z., Beik J. (2022). Fe_3_O_4_@Au Core–Shell Hybrid Nanocomposite for MRI-Guided Magnetic Targeted Photo-Chemotherapy. Lasers Med. Sci..

[160] Xia B., Zhang W., Tong H., Li J., Chen Z., Shi J. (2019). Multifunctional Chitosan/Porous Silicon@Au Nanocomposite Hydrogels for Long-Term and Repeatedly Localized Combinatorial Therapy of Cancer via a Single Injection. ACS Biomater. Sci. Eng..

[161] Namgung H., Jo S., Lee T.S. (2021). Fluorescence Modulation of Conjugated Polymer Nanoparticles Embedded in Poly(N-Isopropylacrylamide) Hydrogel. Polymers.

[162] Bordat A., Boissenot T., Nicolas J., Tsapis N. (2019). Thermoresponsive Polymer Nanocarriers for Biomedical Applications. Adv. Drug Deliv. Rev..

[163] de Solorzano I.O., Prieto M., Mendoza G., Sebastian V., Arruebo M. (2020). Triggered Drug Release from Hybrid Thermoresponsive Nanoparticles Using near Infrared Light. Nanomedicine.

[164] Yavuz M.S., Cheng Y., Chen J., Cobley C.M., Zhang Q., Rycenga M., Xie J., Kim C., Song K.H., Schwartz A.G. (2009). Gold Nanocages Covered by Smart Polymers for Controlled Release with Near-Infrared Light. Nat. Mater..

[165] Song J., Hwang S., Im K., Hur J., Nam J., Hwang S., Ahn G.-O., Kim S., Park N. (2015). Light-Responsible DNA Hydrogel–Gold Nanoparticle Assembly for Synergistic Cancer Therapy. J. Mater. Chem. B.

[166] Zhou J., Wang M., Han Y., Lai J., Chen J. (2020). Multistage-Targeted Gold/Mesoporous Silica Nanocomposite Hydrogel as In Situ Injectable Drug Release System for Chemophotothermal Synergistic Cancer Therapy. ACS Appl. Bio Mater..

[167] Jin R., Yang J., Zhao D., Hou X., Li C., Chen W., Zhao Y., Yin Z., Liu B. (2019). Hollow Gold Nanoshells-Incorporated Injectable Genetically Engineered Hydrogel for Sustained Chemo-Photothermal Therapy of Tumor. J. Nanobiotechnol..

[168] Baseeruddin Alvi S., Rajalakshmi S.R., Begum N., Jogdand A.B., Veeresh B., Rengan A.K. (2021). In Situ Nanotransformable Hydrogel for Chemo-Photothermal Therapy of Localized Tumors and Targeted Therapy of Highly Metastatic Tumors. ACS Appl. Mater. Interfaces.

[169] Jin H., Liu X., Gui R., Wang Z. (2015). Facile Synthesis of Gold Nanorods/Hydrogels Core/Shell Nanospheres for PH and near-Infrared-Light Induced Release of 5-Fluorouracil and Chemo-Photothermal Therapy. Colloids Surf. B Biointerfaces.

[170] Li T., Zhang M., Wang J., Wang T., Yao Y., Zhang X., Zhang C., Zhang N. (2016). Thermosensitive Hydrogel Co-Loaded with Gold Nanoparticles and Doxorubicin for Effective Chemoradiotherapy. AAPS J..

[171] Gonçalves D.P.N., Rodriguez R.D., Kurth T., Bray L.J., Binner M., Jungnickel C., Gür F.N., Poser S.W., Schmidt T.L., Zahn D.R.T. (2017). Enhanced Targeting of Invasive Glioblastoma Cells by Peptide-Functionalized Gold Nanorods in Hydrogel-Based 3D Cultures. Acta Biomater..

[172] Yata T., Takahashi Y., Tan M., Nakatsuji H., Ohtsuki S., Murakami T., Imahori H., Umeki Y., Shiomi T., Takakura Y. (2017). DNA Nanotechnology-Based Composite-Type Gold Nanoparticle-Immunostimulatory DNA Hydrogel for Tumor Photothermal Immunotherapy. Biomaterials.

[173] Li B., Xu Q., Li X., Zhang P., Zhao X., Wang Y. (2019). Redox-Responsive Hyaluronic Acid Nanogels for Hyperthermia- Assisted Chemotherapy to Overcome Multidrug Resistance. Carbohydr. Polym..

[174] Bukowski K., Kciuk M., Kontek R. (2020). Mechanisms of Multidrug Resistance in Cancer Chemotherapy. IJMS.

[175] Sun Y., Wang Q., Chen J., Liu L., Ding L., Shen M., Li J., Han B., Duan Y. (2017). Temperature-Sensitive Gold Nanoparticle-Coated Pluronic-PLL Nanoparticles for Drug Delivery and Chemo-Photothermal Therapy. Theranostics.

[176] Zhong Y., Wang C., Cheng L., Meng F., Zhong Z., Liu Z. (2013). Gold Nanorod-Cored Biodegradable Micelles as a Robust and Remotely Controllable Doxorubicin Release System for Potent Inhibition of Drug-Sensitive and -Resistant Cancer Cells. Biomacromolecules.

[177] Lin W., Yao N., Qian L., Zhang X., Chen Q., Wang J., Zhang L. (2017). PH-Responsive Unimolecular Micelle-Gold Nanoparticles-Drug Nanohybrid System for Cancer Theranostics. Acta Biomater..

[178] Prabaharan M., Grailer J.J., Pilla S., Steeber D.A., Gong S. (2009). Gold Nanoparticles with a Monolayer of Doxorubicin-Conjugated Amphiphilic Block Copolymer for Tumor-Targeted Drug Delivery. Biomaterials.

[179] Zhou G., Xiao H., Li X., Huang Y., Song W., Song L., Chen M., Cheng D., Shuai X. (2017). Gold Nanocage Decorated PH-Sensitive Micelle for Highly Effective Photothermo-Chemotherapy and Photoacoustic Imaging. Acta Biomater..

[180] Parida S., Maiti C., Rajesh Y., Dey K.K., Pal I., Parekh A., Patra R., Dhara D., Dutta P.K., Mandal M. (2017). Gold Nanorod Embedded Reduction Responsive Block Copolymer Micelle-Triggered Drug Delivery Combined with Photothermal Ablation for Targeted Cancer Therapy. Biochim. Biophys. Acta (BBA) Gen. Subj..

[181] Aryal S., Pilla S., Gong S. (2009). Multifunctional Nano-Micelles Formed by Amphiphilic Gold-Polycaprolactone-Methoxy Poly(Ethylene Glycol) (Au-PCL-MPEG) Nanoparticles for Potential Drug Delivery Applications. J. Nanosci. Nanotech..

[182] Lin Y.-C., Perevedentseva E., Lin Z.-R., Chang C.-C., Chen H.-H., Yang S.-M., Lin M.-D., Karmenyan A., Speranza G., Minati L. (2022). Multimodal Bioimaging Using Nanodiamond and Gold Hybrid Nanoparticles. Sci. Rep..

[183] Lin W., Zhang X., Qian L., Yao N., Pan Y., Zhang L. (2017). Doxorubicin-Loaded Unimolecular Micelle-Stabilized Gold Nanoparticles as a Theranostic Nanoplatform for Tumor-Targeted Chemotherapy and Computed Tomography Imaging. Biomacromolecules.

[184] Sarkar S., Konar S., Prasad P.N., Rajput S., Kumar B.N.P., Rao R.R., Pathak A., Fisher P.B., Mandal M. (2017). Micellear Gold Nanoparticles as Delivery Vehicles for Dual Tyrosine Kinase Inhibitor ZD6474 for Metastatic Breast Cancer Treatment. Langmuir.

[185] Sanzhakov M.A., Kudinov V.A., Baskaev K.K., Morozevich G.E., Stepanova D.S., Torkhovskaya T.I., Tereshkina Y.A., Korotkevich E.I., Tikhonova E.G. (2021). Composite Phospholipid-Gold Nanoparticles with Targeted Fragment for Tumor Imaging. Biomed. Pharmacother..

[186] Fogel D.B. (2018). Factors Associated with Clinical Trials That Fail and Opportunities for Improving the Likelihood of Success: A Review. Contemp. Clin. Trials Commun..

[187] Foulkes R., Man E., Thind J., Yeung S., Joy A., Hoskins C. (2020). The Regulation of Nanomaterials and Nanomedicines for Clinical Application: Current and Future Perspectives. Biomater. Sci..

[188] Metselaar J.M., Lammers T. (2020). Challenges in Nanomedicine Clinical Translation. Drug Deliv. Transl. Res..

[189] Begley C.G., Ellis L.M. (2012). Raise Standards for Preclinical Cancer Research. Nature.

[190] Pompili L., Porru M., Caruso C., Biroccio A., Leonetti C. (2016). Patient-Derived Xenografts: A Relevant Preclinical Model for Drug Development. J. Exp. Clin. Cancer Res..

[191] Koga Y., Ochiai A. (2019). Systematic Review of Patient-Derived Xenograft Models for Preclinical Studies of Anti-Cancer Drugs in Solid Tumors. Cells.

[192] Bromma K., Bannister A., Kowalewski A., Cicon L., Chithrani D.B. (2020). Elucidating the Fate of Nanoparticles among Key Cell Components of the Tumor Microenvironment for Promoting Cancer Nanotechnology. Cancer Nano.

[193] Islam R., Maeda H., Fang J. (2022). Factors Affecting the Dynamics and Heterogeneity of the EPR Effect: Pathophysiological and Pathoanatomic Features, Drug Formulations and Physicochemical Factors. Expert Opin. Drug Deliv..

[194] Maeda H. (2015). Toward a Full Understanding of the EPR Effect in Primary and Metastatic Tumors as Well as Issues Related to Its Heterogeneity. Adv. Drug Deliv. Rev..

[195] Park J., Choi Y., Chang H., Um W., Ryu J.H., Kwon I.C. (2019). Alliance with EPR Effect: Combined Strategies to Improve the EPR Effect in the Tumor Microenvironment. Theranostics.

[196] de la Harpe K., Kondiah P., Choonara Y., Marimuthu T., du Toit L., Pillay V. (2019). The Hemocompatibility of Nanoparticles: A Review of Cell–Nanoparticle Interactions and Hemostasis. Cells.

[197] Zhang Y., Bai Y., Jia J., Gao N., Li Y., Zhang R., Jiang G., Yan B. (2014). Perturbation of Physiological Systems by Nanoparticles. Chem. Soc. Rev..

[198] Hante N.K., Medina C., Santos-Martinez M.J. (2019). Effect on Platelet Function of Metal-Based Nanoparticles Developed for Medical Applications. Front. Cardiovasc. Med..

[199] Anderson J.M., Schoen F.J. (2020). In Vivo Assessment of Tissue Compatibility. Biomater. Sci..

